# Ni–Cu bimetallic nanocatalyst stabilized on magnetic nanoparticles for reduction of nitroarenes and one-pot C–N cross-coupling reactions

**DOI:** 10.1039/d5ra04730g

**Published:** 2026-09-28

**Authors:** Mansoore Shahabi, Mohammad Ali Nasseri, Seyyedeh Ameneh Alavi G

**Affiliations:** a Department of Chemistry, Faculty of Sciences, University of Birjand P. O. Box 97175-615 Birjand Iran manaseri@birjand.ac.ir

## Abstract

In the present study, the catalytic reduction of nitroaromatic compounds to amino compounds is evaluated using a heterogeneous recoverable bimetallic nanocatalyst, Fe_3_O_4_@SiO_2_@OERT-Ni-Cu. FE-SEM, TEM, TGA, VSM, XRD, and FT-IR analyses were employed to characterize the structural and morphological properties of the resulting nanocatalyst. The bimetallic nanocatalyst, developed through a cost-effective and environmentally friendly process, demonstrated excellent performance in the reduction of nitro compounds and carbon-nitrogen coupling reactions. Notably, using water as a green solvent, short reaction times (≤2 h), high product yields, and the ease of catalyst recovery and reuse are important advantages of this effective approach. Furthermore, the reusability of the Fe_3_O_4_@SiO_2_@OERT-Ni-Cu bimetallic nanocatalyst was confirmed through seven consecutive runs without any significant loss of catalytic activity in the reduction of nitroaromatic compounds. The high performance of the nanocatalyst is attributed to the synergistic effect between the two metals, nickel and copper.

## Abstract 2

Catalytic reduction of nitroaromatic compounds to amino derivatives using a recoverable Fe_3_O_4_@SiO_2_@OERT-OERTNi-Cu bimetallic nanocatalyst.

## Graphical abstract

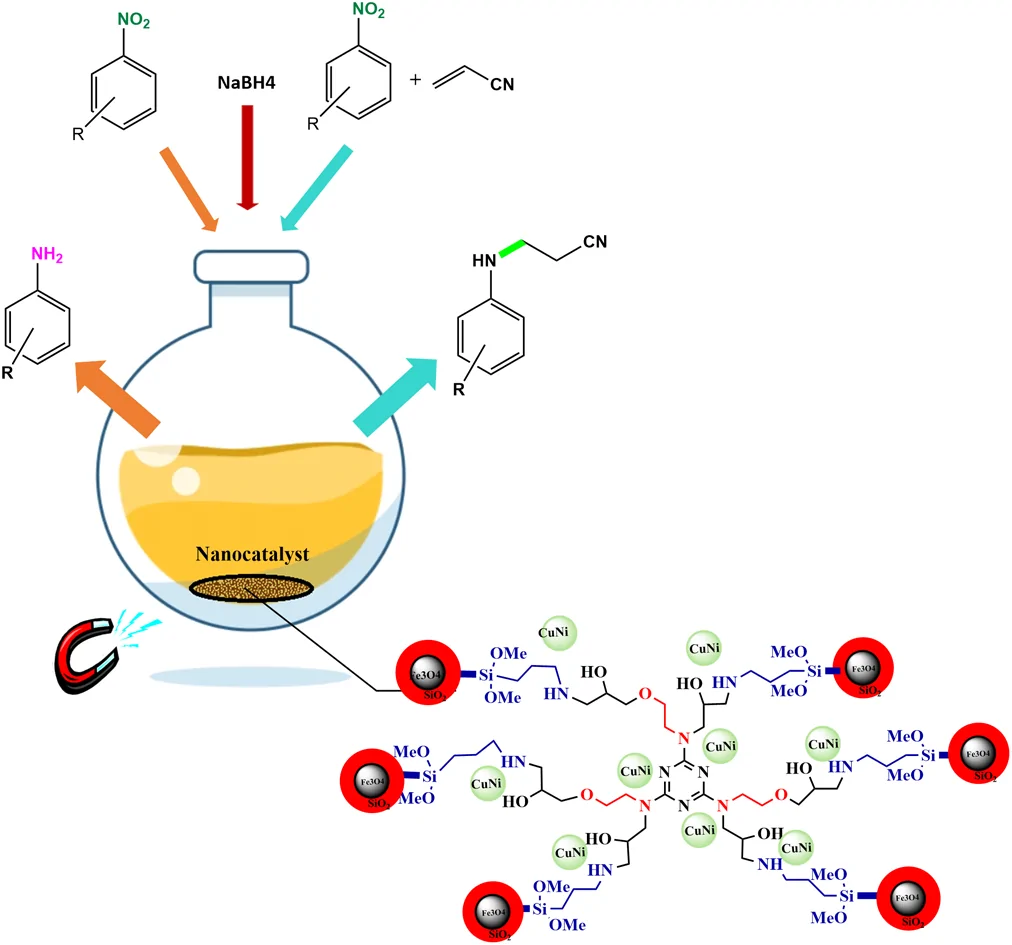

## Introduction

Nowadays, the increase in industrial and human activities, along with the release of pollutants from the textile industry and of organic compounds and their degradation products, has led to environmental water pollution, one of the most critical challenges facing modern society. Aromatic amines are important building blocks in organic chemistry and have widespread applications in the production of dyes, pharmaceuticals, polymers, and agrochemicals.^1–4^ The presence of aromatic nitro compounds as toxic chemicals in water and their associated risks have raised significant concerns among scientific and regulatory communities due to their high stability, water solubility, and carcinogenic effects.^2,5,6^ Therefore, the removal or destruction of these toxic compounds is of great importance.

With the development of nanotechnology, several methods-including electrochemical oxidation,^7^ surface adsorption,^8^ biological decomposition,^9^ and photocatalytic degradation^10,11^ have been employed to eliminate or degrade many persistent chemical compounds. Among these methods, the catalytic reduction of nitroaromatic compounds to amino compounds has garnered significant attention due to its high efficiency, scalability, economic viability, and sustainability.^12,13^ By converting nitroarenes to aromatic amines, a wide range of functionalized aromatic compounds can be synthesized, enabling the production of diverse chemical structures.^14,15^ Various substances have been used as catalysts for the reduction of toxic aromatic nitro compounds, exhibiting different levels of activity. Over the past decades, well-designed and effective heterogeneous catalysts have attracted increasing interest due to the remarkable features, including easy availability, thermal stability, and high surface area.^16,17^ Recently, magnetic nanoparticles (MNPs) have been employed as suitable heterogeneous catalysts for the reduction of aromatic amine compounds.^18^ MNPs-based heterogeneous catalysts can be easily separated from reaction systems using an external magnet.^17^ Specifically, bimetallic catalysts offer exceptional catalytic capabilities due to the synergistic combination of two distinct metals.^19–22^ Gosh *et al.*^23^ investigated the catalytic activity of Pt–Ni bimetallic MNPs for the reduction of aromatic nitro compounds, finding enhanced catalytic activity and corrosion resistance in the reduction of 4-nitrophenol assisted by sodium borohydride (NaBH_4_). In another study, high catalytic activity of bimetallic MNPs for the selective reduction of nitroaromatics was attributed to strong interaction between Au and Ag species.^24^ Additionally, Gholinejad *et al.*^25^ explored the use of magnetic bimetallic nanocatalysts as an efficient route for the reduction of nitroarenes, demonstrating no significant decrease in catalytic activity compared to single-metal analogs. Currently, the design of nanocatalysts based on low-cost, readily available metals such as copper (Cu) and nickel (Ni) is being actively pursued. Literature reports indicate that nickel and copper are commonly used in nanocatalysts due to their unique properties, which make them effective catalysts for various chemical reactions.^26,27^ Synergistic interactions between Ni and Cu metals can enhance both the efficiency and selectivity of the reduction processes.^17^ In this study, we report the performance of a bimetallic nickel-copper magnetic recoverable nanocatalyst (Scheme 1) in the reduction of nitro compounds. This nanocatalyst provides a high surface area that facilitates interaction between the reactants (nitroarenes and NaBH_4_) and promotes catalytic activity during the reduction reaction.

**Scheme 1:**
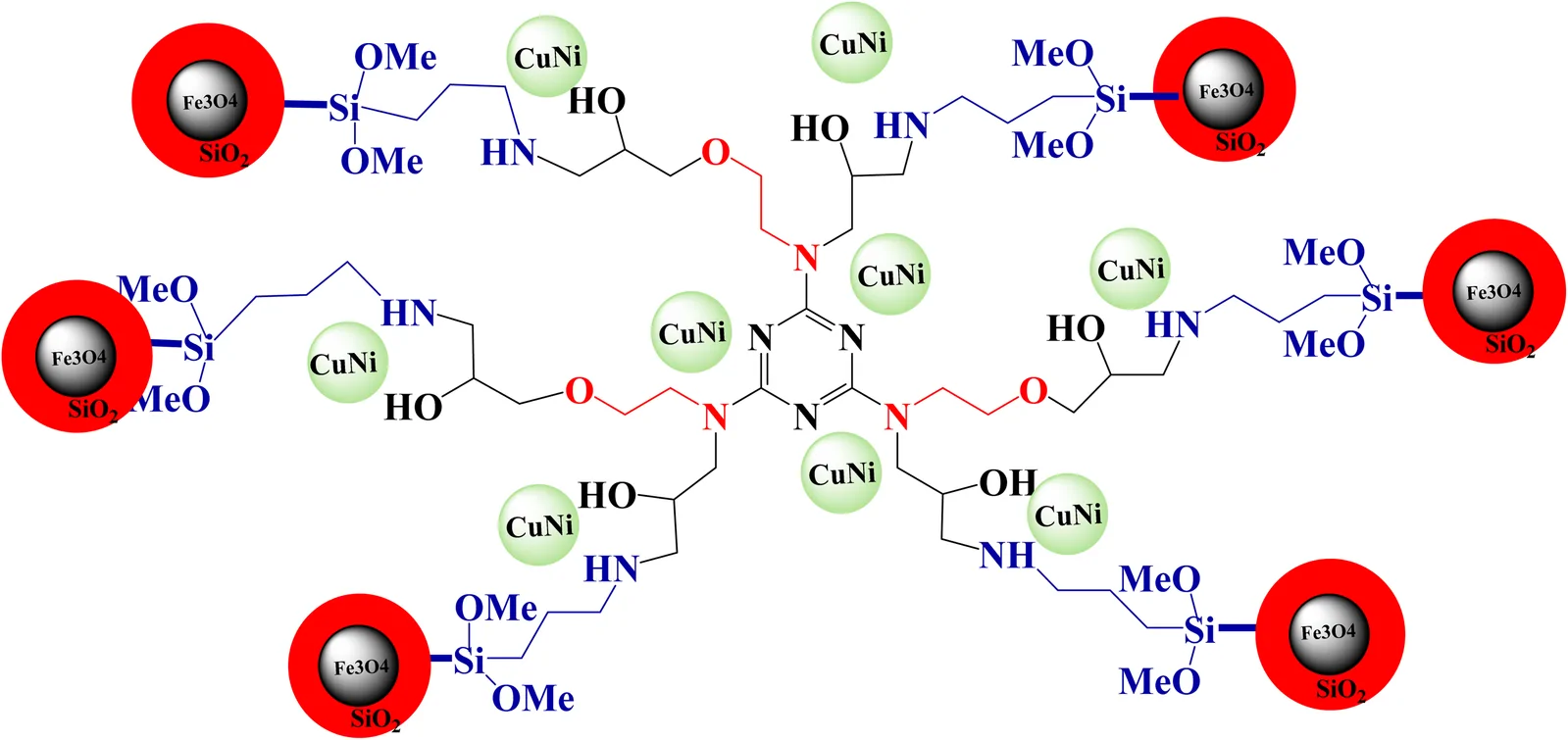
Fe_3_O_4_@SiO_2_@OERT-Ni-Cu nanocatalyst structure.

## Experimental procedure

### Materials and instruments

All chemicals were purchased from Sigma-Aldrich and Fluca suppliers and used without further purification. Fourier transform infrared (FT-IR) spectra were recorded on a JASCO FT/IR 4600 spectrometer. Field emission scanning electron microscopy (FE-SEM) and energy-dispersive X-ray spectroscopy (EDS) analyses were performed using a FEI Quanta- 200 SEM and a JEOL 7600F FE-SEM apparatus. Transmission electron microscopy (TEM) images were obtained using a Philips EM208S microscope operated at 100 kV. Powder X-ray diffraction (XRD) patterns were recorded on a Rigaku SmartLab instrument. Thermogravimetric analysis (TGA) of the samples was conducted under a nitrogen atmosphere over a temperature range of 25–1000 °C with a heating rate of 10 °C min^−1^ (Q600 model, TA Instruments, USA). Thin layer chromatography (TLC) was used to monitor the progress of the reactions. Magnetization measurements of the samples were performed at room temperature using a vibrating sample magnetometer (VSM, Lake Shore Cryotronics 7407).

### Preparation of Fe_3_O_4_@SiO_2_

Core–shell Fe_3_O_4_@SiO_2_ nanoparticles were synthesized following a previously reported protocol.^17^ Briefly, FeCl_3_·6H_2_O (4.809 mmol, 1.3 g) and FeCl_2_·4H_2_O (4.526 mmol, 0.9 g) were dissolved in 300 mL of distilled water with vigorous mechanical stirring at 50 °C for 30 minutes. Then, NaOH (50 mL, 10% by weight) was slowly added to the solution, maintaining the pH at 10. The reaction mixture was stirred at 50 °C for 2 hours. The black magnetite was separated by applying an external magnetic field, rinsed several times with water and ethanol, and dried under vacuum at 80 °C for 10 hours. For the synthesis of Fe_3_O_4_@SiO_2_, 0.5 g of the synthesized Fe_3_O_4_ nanoparticles was dispersed in a mixture containing ethanol (20 mL) and deionized water (20 mL) for 30 minutes. Then, a mixture of tetraethyl orthosilicate (1 mL) in ethanol (10 mL) and NaOH (0.5 mL, 10% by weight) was added dropwise, and the resulting mixture was stirred for 2 hours. The Fe_3_O_4_@SiO_2_ nanoparticles were collected using an external magnet, washed with distilled water and ethanol, and dried in a vacuum oven at 80 °C for 10 hours.

### Preparation of Fe_3_O_4_@SiO_2_-APTES

Initially, 1 g of Fe_3_O_4_@SiO_2_ was dispersed in 20 mL of dry toluene for 1 hour. Then, a mixture containing 1 mL of 3-aminopropyltriethoxysilane (APTES), 10 mL of dry toluene, and 0.3 mL of Et_3_N as a base was added dropwise to the suspension. The resulting mixture was refluxed under an argon atmosphere at 110 °C for 48 hours. The obtained Fe_3_O_4_@SiO_2_-NH_2_ was separated using an external magnet, washed with toluene, and dried under vacuum.

### Synthesis of OERT (open epoxy resin triazine)

In the first step, we condensed 6.673 × 10^−3^ mol of ethanolamine with 3.951 × 10^−3^ mol of 2,4,6-trichloro-1,3,5-triazine using methanol as the solvent under reflux and magnetic stirring for 48 hours (Scheme 2). In the second step, we added 7.862 × 10^−3^ mol of epichlorohydrin to trihydroxy triethanamine triazine under magnetic stirring and refluxed the mixture for 4 hours. Methanol was then removed using a rotary evaporator.^28^

**Scheme 2:**
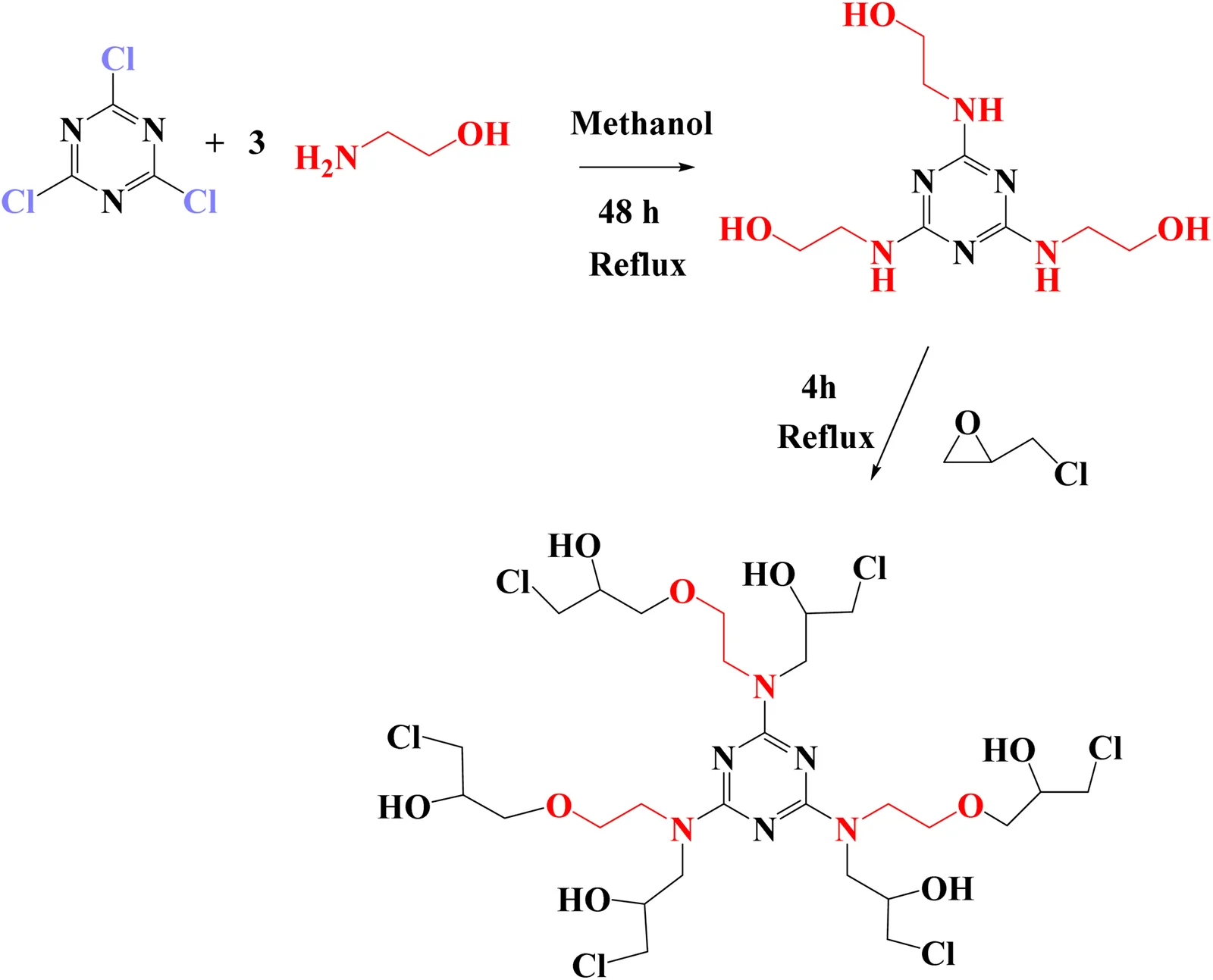
Synthesis of OERT.

### Preparation of Fe_3_O_4_@SiO_2_@OERT

The OERT (0.2 g) was added to a suspension of Fe_3_O_4_@SiO_2_-NH_2_ (0.6 g) in ethanol (20 mL). The reaction mixture was stirred at 60 °C for 6 hours. Fe_3_O_4_@SiO_2_@OERT was isolated using an external magnet, washed with ethanol, and dried at 60 °C for 12 hours.

### Preparation of copper(ii) and nickel(ii) immobilized on Fe_3_O_4_@SiO_2_@OERT

To prepare the Fe_3_O_4_@SiO_2_@OERT-Ni-Cu structure, 0.5 g of the Fe_3_O_4_@SiO_2_@OERT was dispersed in 40 mL of ethanol, and the suspension was stirred for 30 minutes at room temperature. Then, 0.05 g (0.212 mmol) of Cu(OAC)_2_·3H_2_O and 0.05 g (0.200 mmol) of Ni (OAC)_2_·4H_2_O were added to the mixture. The reaction mixture was refluxed at 80 °C for 20 hours. Afterwards, the precipitate was filtered, washed four times with ethanol, and dried.

### Typical procedure for the reduction of nitroarenes to arylamines catalyzed by Fe_3_O_4_@SiO_2_@OERT-Ni-Cu

A reaction mixture containing nitro compounds (0.5 mmol), sodium borohydride (1.5 mmol), and Fe_3_O_4_@SiO_2_@OERT-Ni-Cu catalyst (30 mg; 0.3 mol% Ni, 0.33 mol% Cu) in water (3 mL) was stirred at 70 °C for an appropriate duration. The progress of the reaction was monitored by TLC. Upon completion, the catalyst was separated from the reaction mixture using a magnet. The product was extracted with ethyl acetate, and the organic layer was dried over anhydrous sodium sulfate (Na_2_SO_4_) and concentrated to yield the crude product. Pure products were obtained by silica gel column chromatography using an *n*-hexane : EtOAc (5 : 15) solvent system.

### Typical procedure for the one-pot reductive C–N cross-coupling of nitroarenes catalyzed by Fe_3_O_4_@SiO_2_@OERT-Ni-Cu

To perform a one-pot reductive C–N cross-coupling reaction, a mixture of nitro compounds (0.5 mmol) in H_2_O (3 mL) was prepared. Then, Fe_3_O_4_@SiO_2_@OERT-Ni-Cu (30 mg, 0.3 mol% Ni, 0.33 mol% Cu) and NaBH4 (1.5 mmol) were added, and the mixture was stirred at 70 °C. After completion of the reaction (monitored by thin-layer chromatography), acrylonitrile (1.2 mmol) was added, and stirring continued at 70 °C. The product was extracted with ethyl acetate, and the organic layer was dried over Na_2_SO_4_ and concentrated to yield the crude product. Pure products were obtained by silica gel column chromatography using *n*-hexane : EtOAc (8 : 12) as the eluent.

## Results and discussion

The synthesis overview of the new and recyclable bimetallic nanocatalyst is presented in Scheme 3. Several physicochemical techniques, including FT-IR, XRD, VSM, EDS, TGA, TEM, and FE-SEM, were employed to characterize the synthesized nanocatalyst.

**Scheme 3:**
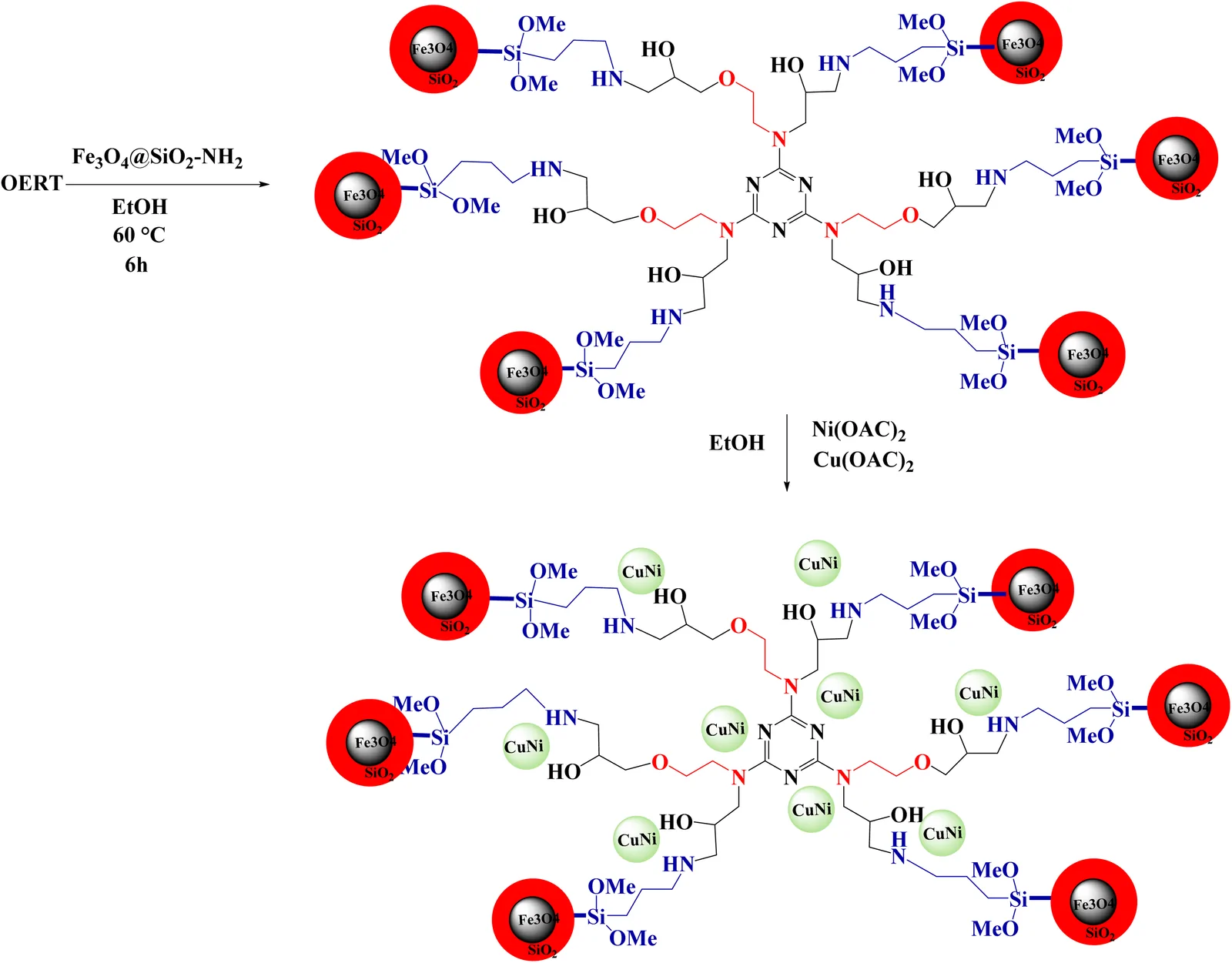
Synthesis route for a new recyclable Fe_3_O_4_@SiO_2_@OERT-Ni-Cu nanocatalyst.

### Catalyst characterizations

FT-IR absorption spectra for different stages of Fe_3_O_4_@SiO_2_@OERT-Ni-Cu synthesis are shown in Fig. 1. In the spectrum of the Fe_3_O_4_ magnetic nanoparticles (Fig. 1a), the stretching vibration of the Fe–O bond appears at 521 cm^−1^. The asymmetric Si–O–Si stretching vibrations appear at 1095 cm^−1^, and the absorption band at 794 cm^−1^ corresponds to the symmetric Si–O–Si stretching vibrations, confirming the successful coating of silica on the surface of the Fe_3_O_4_ nanoparticles (Fig. 1b). The observation of absorption bands for aliphatic C–H and N–H bonds at 2810–2900 cm^−1^ and 3350 cm^−1,^ respectively, illustrates the successful bonding of APTES to the magnetic nanoparticle surface (Fig. 1c). The FT-IR spectrum of synthesized OERT (Fig. 1d) shows an absorption band at 3250 cm^−1^, attributed to OH stretching vibrations. The band at 2900 cm^−1^ corresponds to aliphatic C–H, while the bands at 1670 and 1359 cm^−1^ indicate the vibrations of C=N and aromatic C–N, respectively. The C–O bond vibrations are observed at 1070 cm^−1^. These bands confirm the successful synthesis of the OERT compound. Fig. 1e corresponds to the synthesis of Fe_3_O_4_@SiO_2_@OERT. The absorption band at 3340 cm^−1^ in curve e can be assigned to N–H bond vibrations, confirming the formation of OERT on the nanoparticle surface. The bands at 3146, 1370, and 1647 cm^−1^ are characteristic of O–H stretching, C–N, and C=N bonds of triazine, respectively, indicating the presence of OERT on the nanoparticle surface. As shown in Fig. 1f, the Ni and Cu species were successfully immobilized onto the magnetic Fe_3_O_4_@SiO_2_@OERT support. The FT-IR spectrum of the resulting catalyst displays the characteristic absorption bands corresponding to the different components of the catalyst, confirming the successful incorporation of the metal species onto the functionalized magnetic support.

**Fig. 1:**
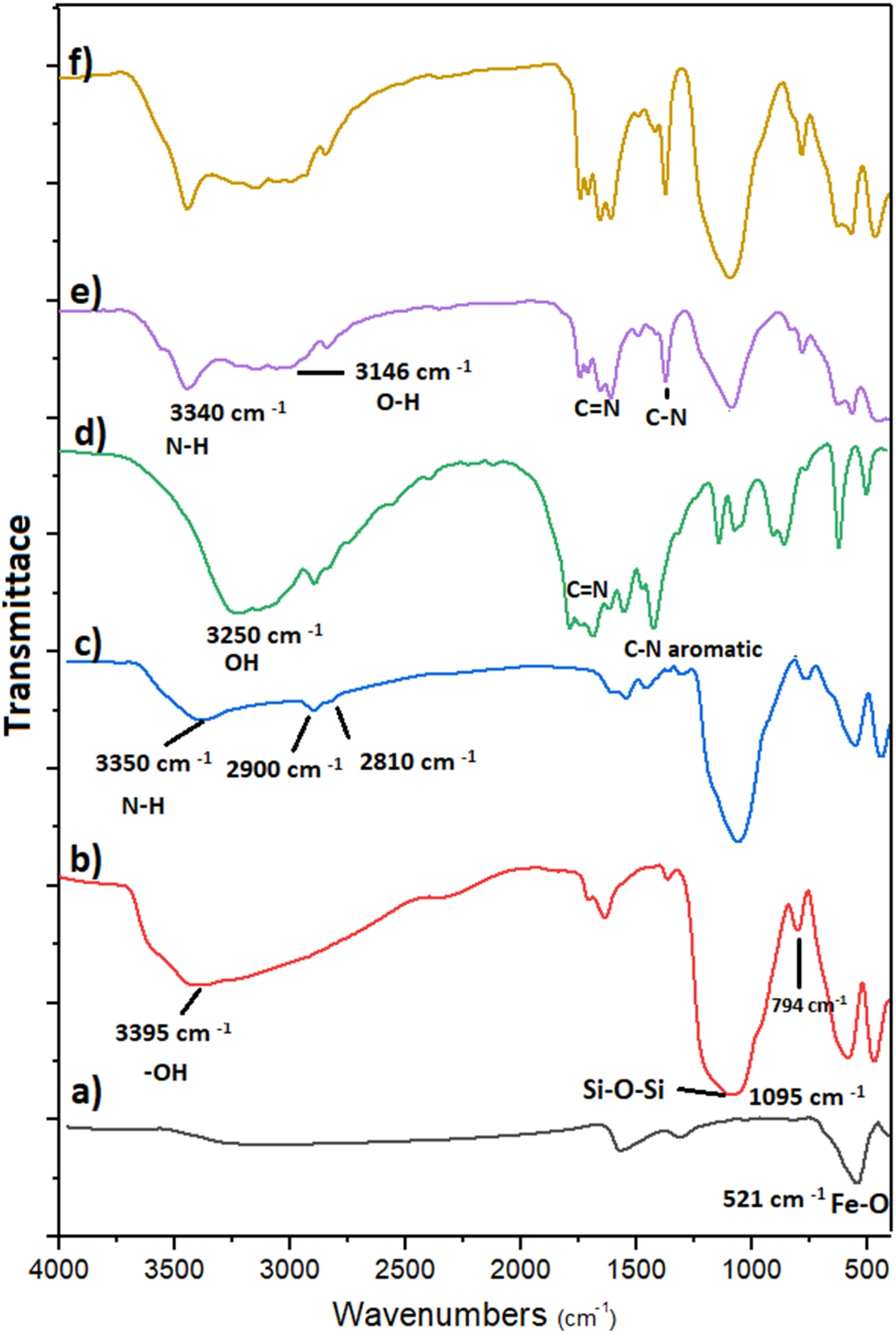
FT-IR analysis for the characterization of (a) Fe_3_O_4_, (b) Fe_3_O_4_@SiO_2_, (c) Fe_3_O_4_@SiO_2_-NH_2_, (d) OERT, (e) Fe_3_O_4_@SiO_2_-OERT, and (f) Fe_3_O_4_@SiO_2_@OERT-Ni-Cu nanocatalyst, respectively.

The XRD patterns of Fe_3_O_4_ nanoparticles and the Fe_3_O_4_@SiO_2_@OERT-Ni-Cu nanocatalyst are shown in Fig. 2. As observed, the main peaks at 2*θ* = 30.29°, 35.59°, 43.39°, 54.19°, 57.44°, and 63.14° correspond to the (220), (311), (400), (422), (511), and (440) planes, respectively, confirming the crystalline structure of Fe_3_O_4_ nanoparticles. Comparing the XRD patterns of the synthesized nanocatalyst and Fe_3_O_4_ nanoparticles reveals a decrease in peak intensity of the Fe_3_O_4_@SiO_2_@OERT-Ni-Cu nanocatalyst, which can be attributed to the coating of amorphous SiO_2_ and the OERT complex on the surface of Fe_3_O_4_ nanoparticles. Additionally, a strong peak appears at 2*θ* = 27.79° in the XRD pattern of the Fe_3_O_4_@SiO_2_@OERT-Ni-Cu catalyst, indicating the crystallinity of the OERT coating.

**Fig. 2:**
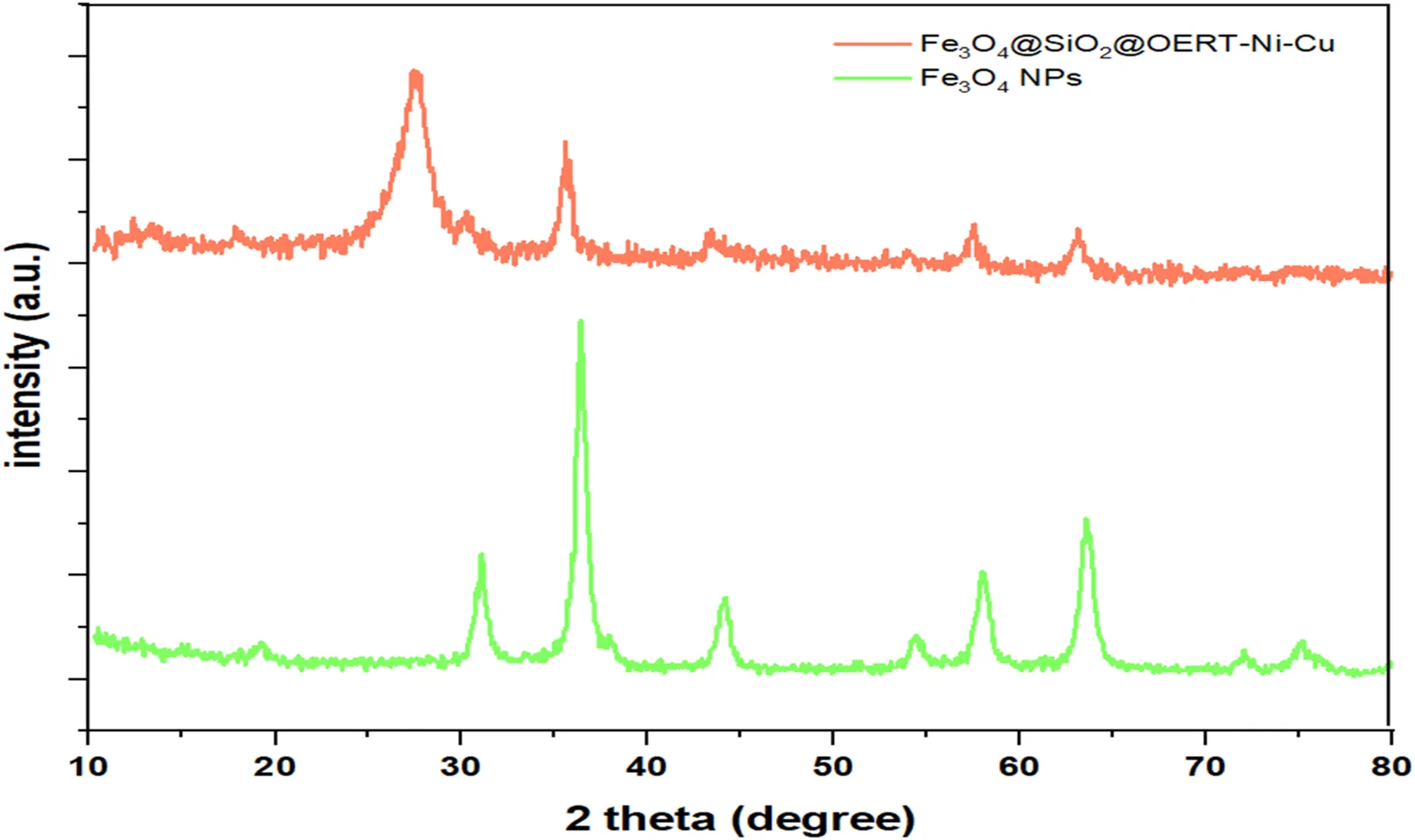
XRD pattern of Fe_3_O_4_ NPs and Fe_3_O_4_@SiO_2_@OERT-Ni-Cu nanocatalyst.

Fig. 3 illustrates the magnetic properties of Fe_3_O_4_ nanoparticles and the Fe_3_O_4_@SiO_2_@OERT-Ni-Cu nanocatalyst as determined by VSM analysis. The magnetic curves for Fe_3_O_4_ NPs and the Fe_3_O_4_@SiO_2_@OERT-Ni-Cu nanocatalyst show saturation magnetization values of 64.79 and 24 amu g^−1^, respectively, indicating the superparamagnetic nature of both materials. Despite the reduction in the amount of magnetization of the synthesized nanocatalyst, it can still be separated from the reaction medium using a magnet.

**Fig. 3:**
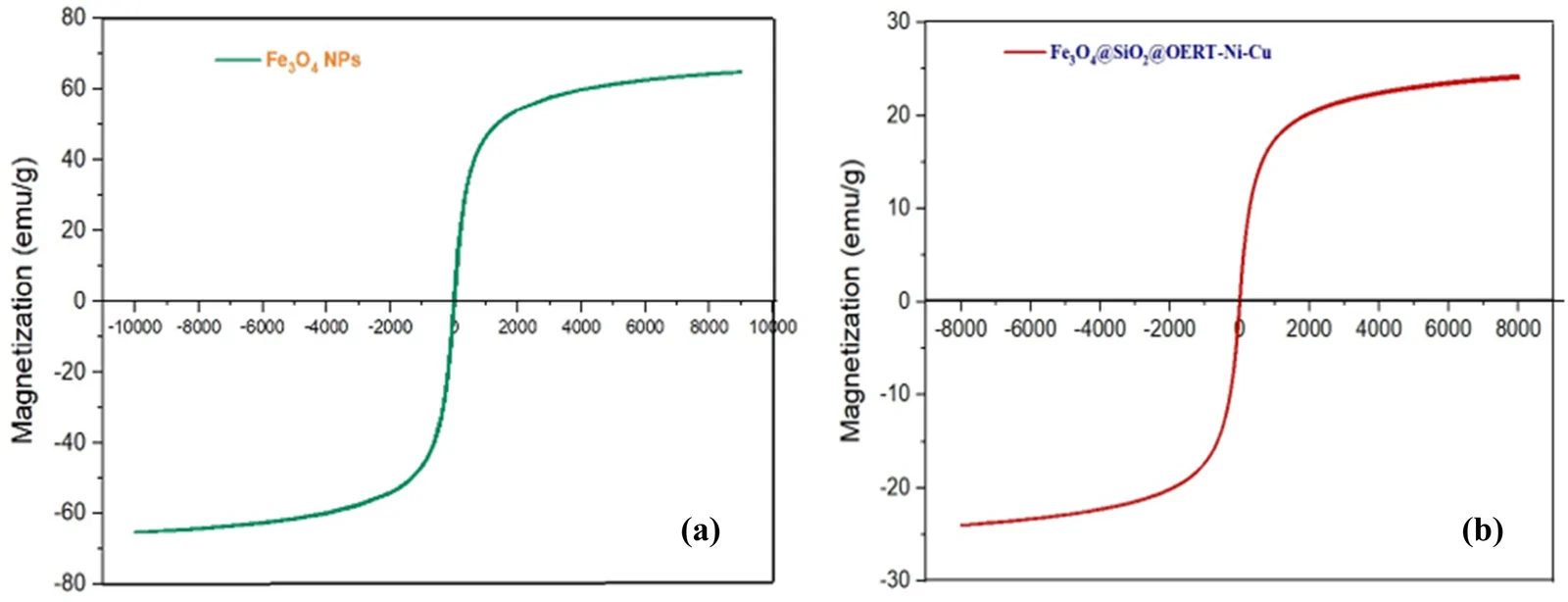
VSM pattern of (a) Fe_3_O_4_ NPs and (b) Fe_3_O_4_@SiO_2_@OERT-Ni-Cu nanocatalyst.

TGA analysis was conducted to investigate the thermal behavior of the Fe_3_O_4_@SiO_2_@OERT-Ni-Cu nanocatalyst, as shown in Fig. 4. The TGA curve indicates a total weight loss of 32.7% over the temperature range of 30 to 1000 °C, occurring in two distinct stages. The first weight loss (approximately 10%) occurs between 30 and 280 °C and corresponds to the removal of physically adsorbed solvents and surface hydroxyl groups. The second weight loss stage, which is about 22.7% and occurs between 280 and 1000 °C, is attributed to the decomposition of various organic components on the surface of the Fe_3_O_4_ nanoparticles.

**Fig. 4:**
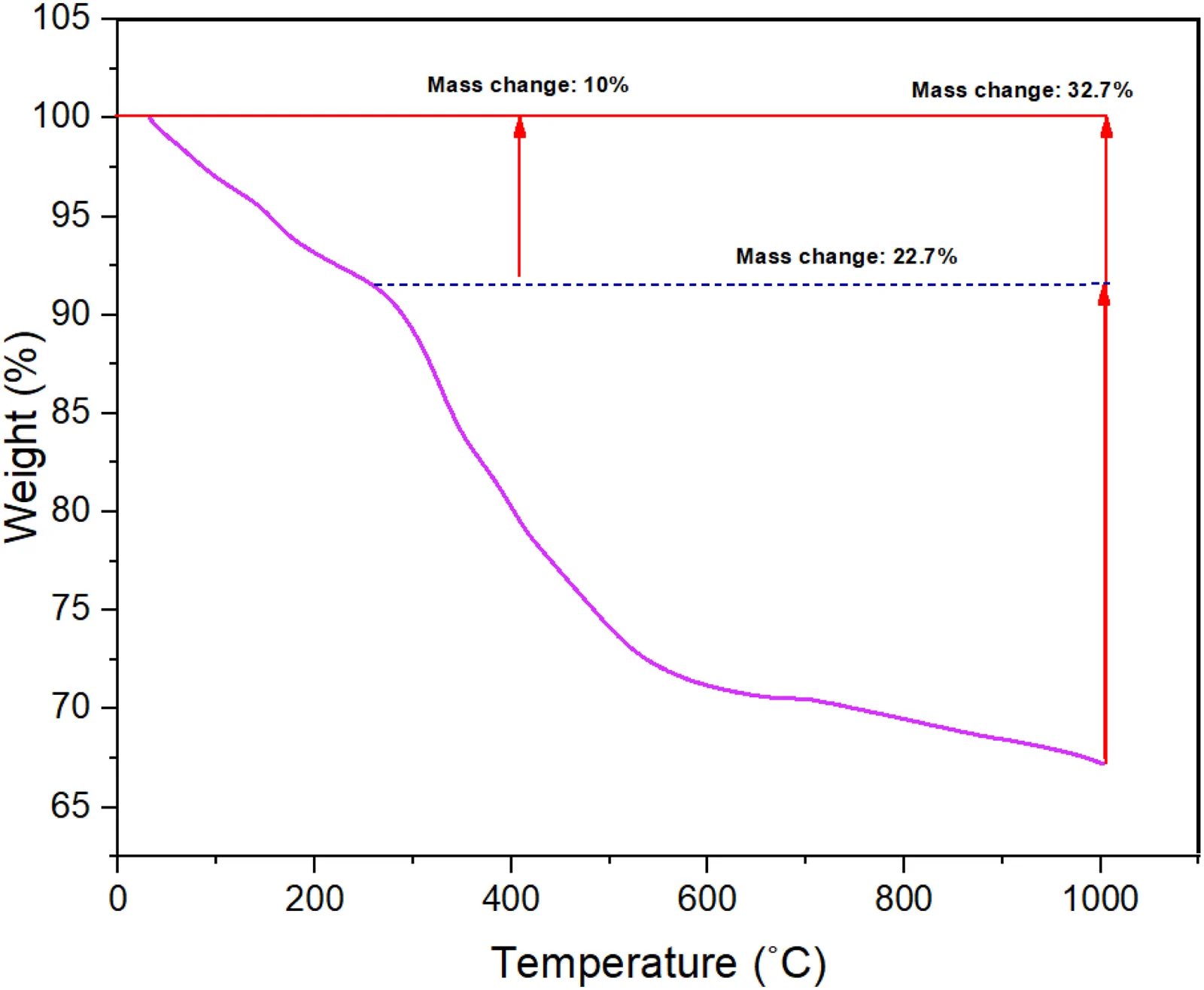
TGA curve of Fe_3_O_4_@SiO_2_@OERT-Ni-Cu nanocatalyst.

The composition of the Fe_3_O_4_@SiO_2_@OERT-Ni-Cu nanocatalyst particles was analyzed using EDS, as shown in Fig. 5. This analysis reveals the presence of carbon, nitrogen, oxygen, iron, copper, and nickel elements within the Fe_3_O_4_@SiO_2_@OERT-Ni-Cu nanocatalyst. The two metallic elements, nickel and copper, are present at percentages of 0.4% and 0.56%, respectively, indicating that the synthesized nanocatalyst is bimetallic.

**Fig. 5:**
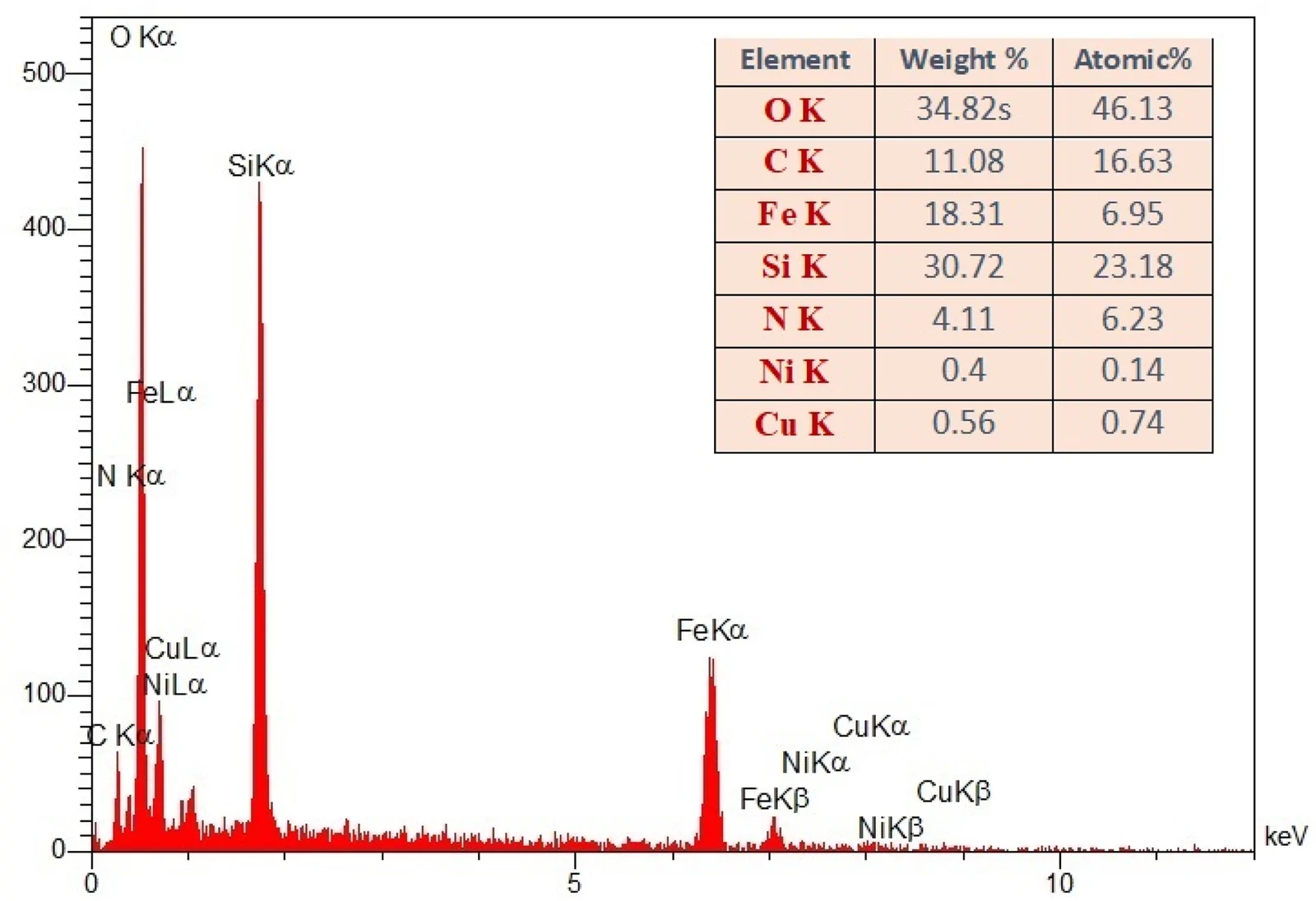
EDS analysis of Fe_3_O_4_@SiO_2_@OERT-Ni-Cu nanocatalyst.

ICP analysis of the Fe_3_O_4_@SiO_2_@OERT-Ni-Cu complex nanocomposite reveals 0.72 wt% Cu and 0.6 wt% Ni in the catalyst.

The shape and size of Fe_3_O_4_@SiO_2_@OERT-Ni-Cu nanocatalyst particles are shown in the TEM images in Fig. 6a and b. The average particle size observed in the TEM images is approximately 20 nm. As illustrated in Fig. 6, the nanoparticles are dispersed within the Fe_3_O_4_@SiO_2_@OERT-Ni-Cu nanocatalyst matrix as spherical particles. Fig. 6c presents the surface morphology of the Fe_3_O_4_@SiO_2_@OERT-Ni-Cu nanocatalyst through FE-SEM images. As seen in Fig. 6c, spherical particles are uniformly distributed across the surface of the nanocatalyst.

**Fig. 6:**
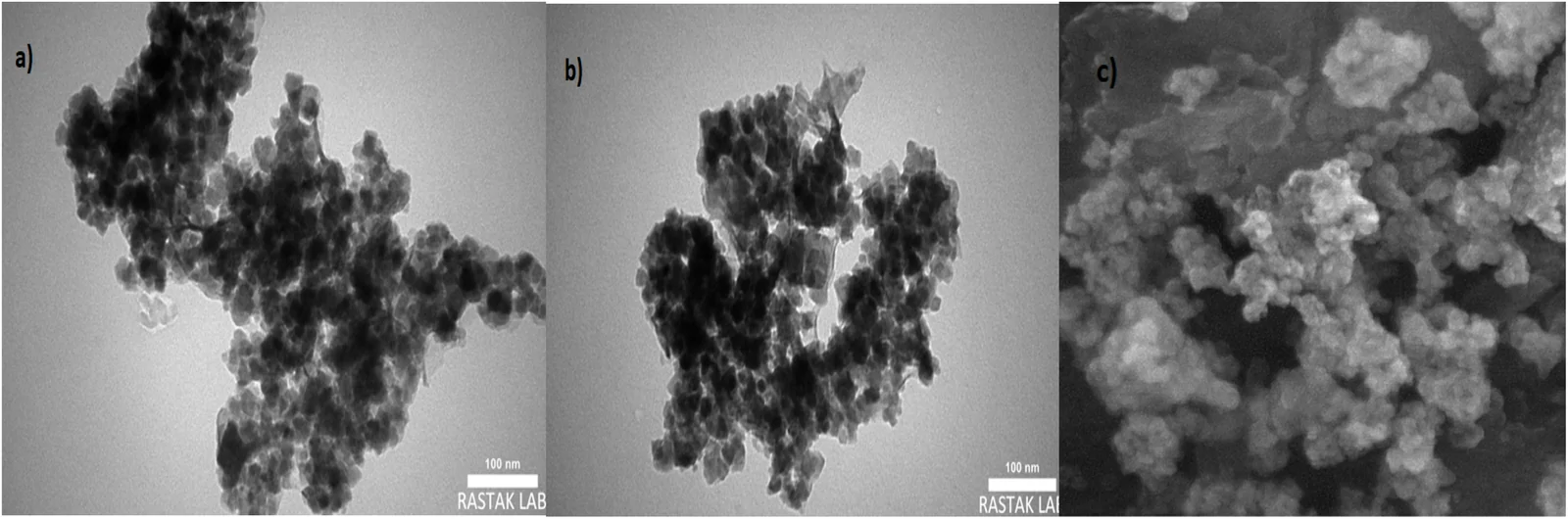
(a) and (b) TEM and (c) FE-SEM images of Fe_3_O_4_@SiO_2_@OERT-Ni-Cu nanocatalyst.

### Catalytic activity

#### Reduction of nitroarenes with NaBH_4_/Fe_3_O_4_@SiO_2_@OERT-Ni-Cu system

After synthesizing and characterizing the new Fe_3_O_4_@SiO_2_@OERT-Ni-Cu nanocatalyst, we evaluated its catalytic efficiency in the reduction of aromatic nitro compounds and C–N cross-coupling reactions, as illustrated in Scheme 4. To optimize the reaction conditions, the reduction of nitrobenzene was selected as the model reaction, and the effects of solvent, temperature, amount of NaBH_4_, and catalyst loading were systematically investigated (Table 1).

**Scheme 4:**
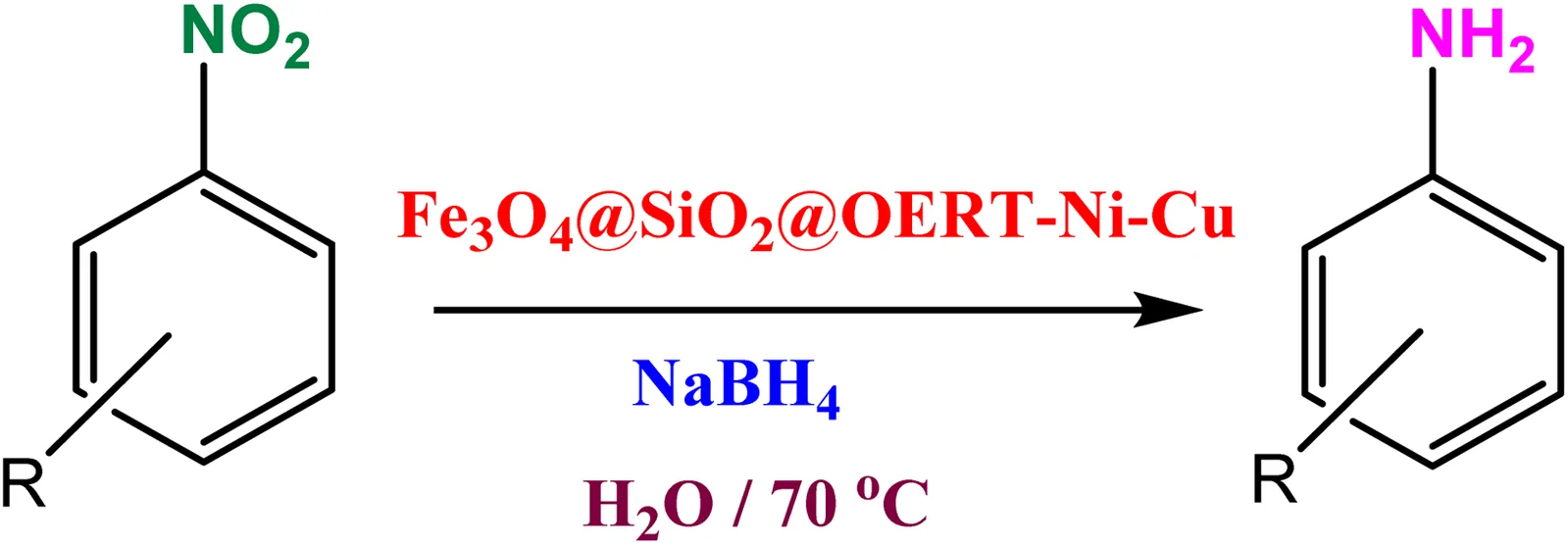
Reduction of nitroarenes using NaBH4 catalyzed by Fe_3_O_4_@SiO_2_@OERT-Ni-Cu nanocatalyst.

**Table 1:** Optimization of the reduction reaction parameters of PhNO_2_ to aniline with NaBH_4_ catalyzed by Fe_3_O_4_@SiO_2_@OERT-Ni-Cu nanocatalyst^a^

Entry	Cat. amount (mg)	NaBH_4_ (mmol)	Solvent	Tem. (°C)	Conversion (%)
1	10	1	Ethanol	70	50
2	10	1	Water	70	60
3	10	1	Ethyl acetate	70	No reaction
4	10	1	DMF	70	No reaction
5	10	1	DMSO	70	No reaction
6	20	1	Water	70	70
7	30	1	Water	70	93
8	30	1.5	Water	70	98
9	30	2	Water	70	99
10	30	3	Water	70	99
11	30	1.5	Water	50	90
12	30	1.5	Water	r.t	45

a Reaction conditions: nitrobenzene (0.5 mmol), NaBH_4_ (1.5 mmol), cat. (0.03 g), H_2_O (3 mL), 70 °C, 1 h.

The reaction is conducted in the presence of various protic and aprotic solvents, including ethanol, H_2_O, ethyl acetate, DMF, and DMSO. The reaction yield indicates that H_2_O is the most suitable solvent for reducing nitro compounds. The reduction reaction is performed at room temperature, 50 °C, and 70 °C, with the highest efficiency observed at 70 °C. The effect of the amount of NaBH_4_ on reaction efficiency is also investigated, using 1, 1.5, 2, and 3 mmol of NaBH_4_ in the reduction of nitro compounds. The results show that 1.5 mmol of NaBH_4_ achieves the highest conversion. The best result corresponds to 30 mg of the Fe_3_O_4_@SiO_2_@OERT-Ni-Cu nanocatalyst. Thus, the optimal conditions are: a temperature of 70 °C, H_2_O as the solvent, and 30 mg of the catalyst (0.3 mol% Ni, 0.33 mol% Cu) (Table 1, entry 8).

Under these conditions, the efficiency and chemoselectivity of the Fe_3_O_4_@SiO_2_@OERT-Ni-Cu nanocatalyst in the reduction of aromatic nitro compounds are evaluated using a variety of aromatic nitro compounds, as presented in Table 2. Nitrobenzene and the dinitrobenzoic acid derivative were selectively transformed into 2a and 2b in excellent yields of 99%, while the carboxylic acid group remained unaffected. Iodo-, chloro-, cyano-, and hydroxy-substituted nitroarenes produced 2e, 2h, 2i, and 2j in yields of 75%, 65%, 90%, and 95%, respectively, showing good compatibility with these functional groups and a clear preference for reducing the nitro group. In contrast, substrates containing carbonyl groups displayed lower chemoselectivity. For example, nitrobenzaldehyde gave 2c through selective nitro reduction, but further reduction of the aldehyde produced 2d in a 40 : 60 ratio. Likewise, nitroacetophenone resulted in 2f and 2g in a 50 : 50 ratio, suggesting competing reduction routes. With the nitrobenzoyl chloride substrate, 2k was formed in 70% yield, along with conversion of the acid chloride group into a carboxylic acid. Overall, the catalyst shows good chemoselectivity for NO_2_ reduction—especially when carboxylic acid, halogen, cyano, and hydroxy groups are present—although reactive carbonyl functionalities can still undergo additional reactions under the conditions.

**Table 2:** Reduction of various nitroarene compounds catalyzed by Fe_3_O_4_@SiO_2_@OERT-Ni-Cu nanocatalyst^a^

Entry	Substrate	Product	Product no.	Time (h)	Yield^b^ (%)
1	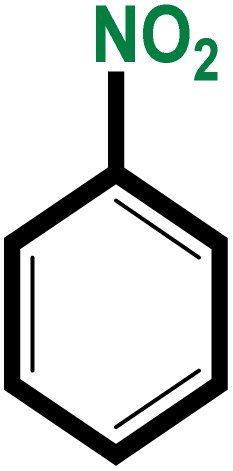	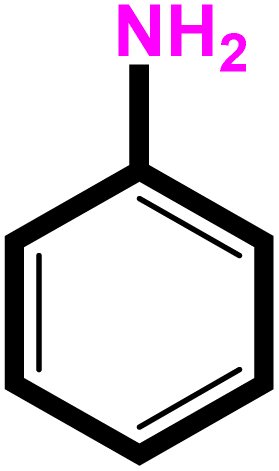	2a	1	99
2	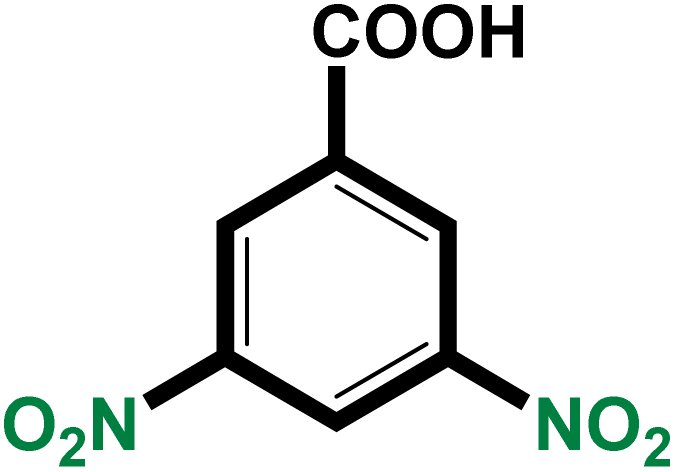	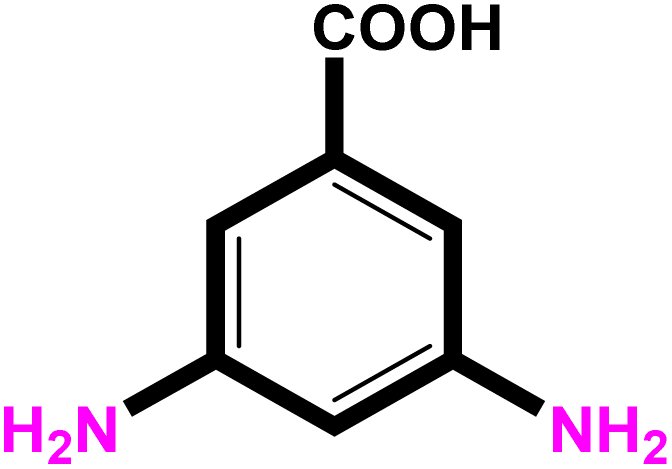	2b	1	99
3	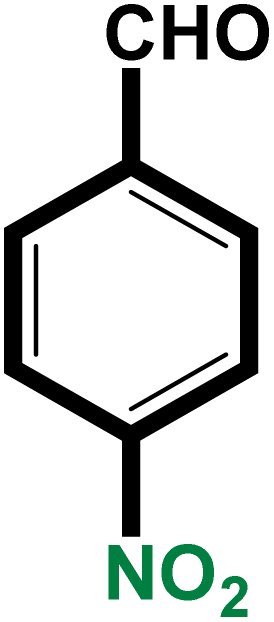	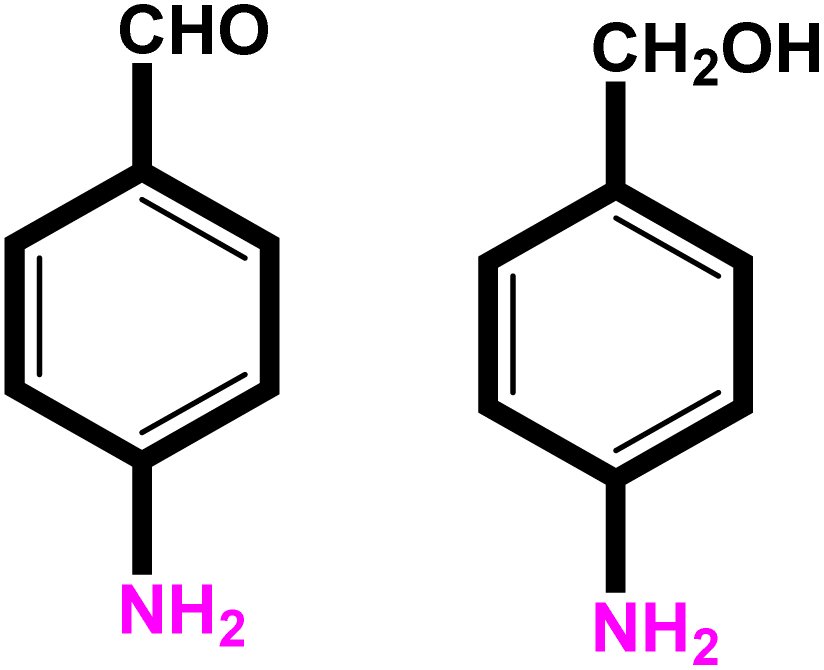	2c, 2d	1	40(2c)–60(2d)
					Two products
4	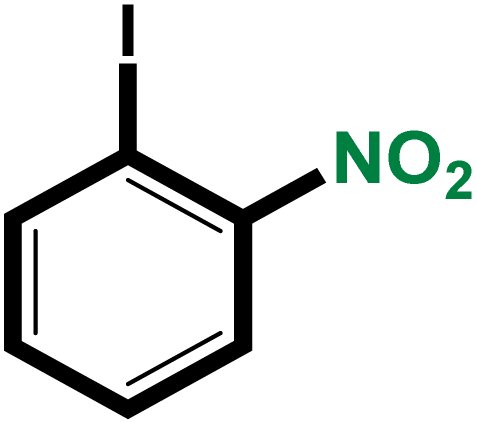	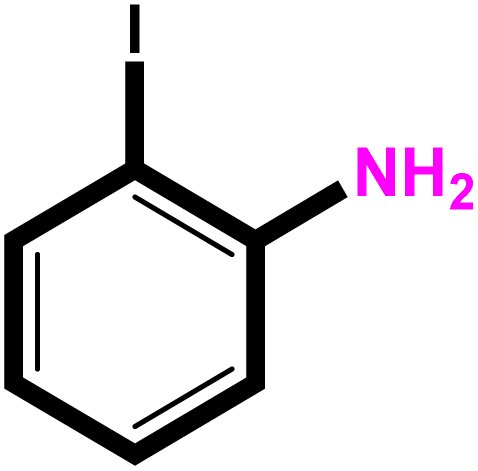	2e	1	75
5	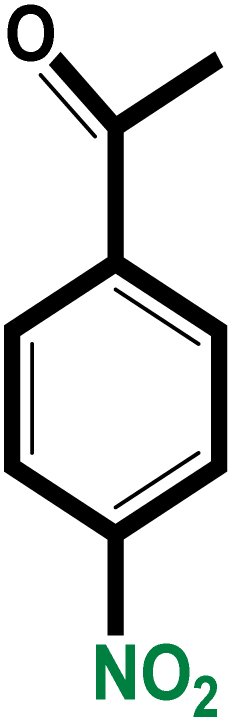	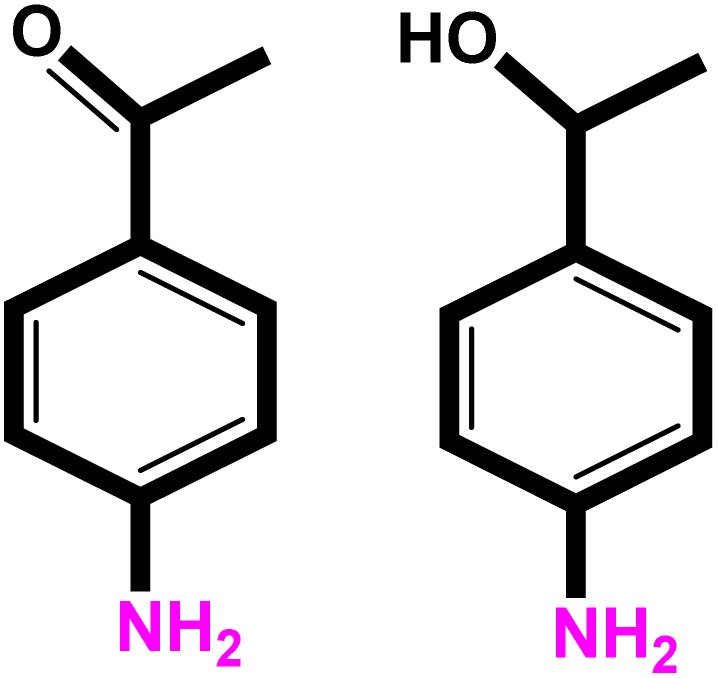	2f, 2g	1	50–50
					Two products
6	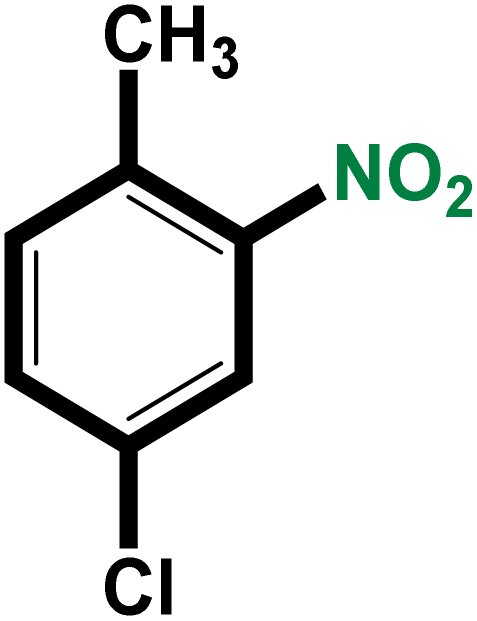	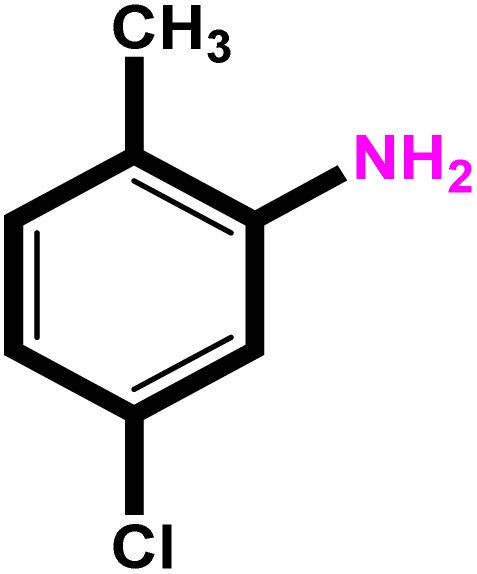	2h	1	65
7	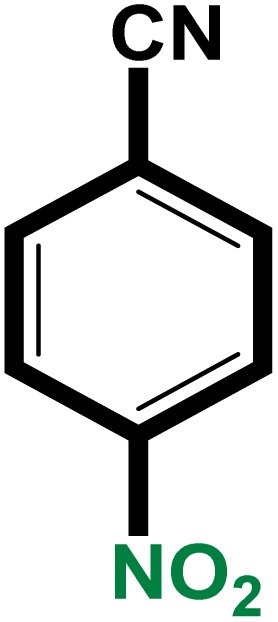	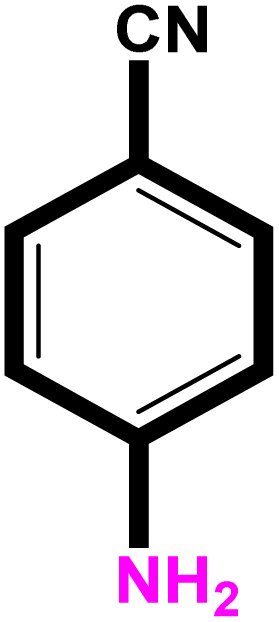	2i	1.5	90
8	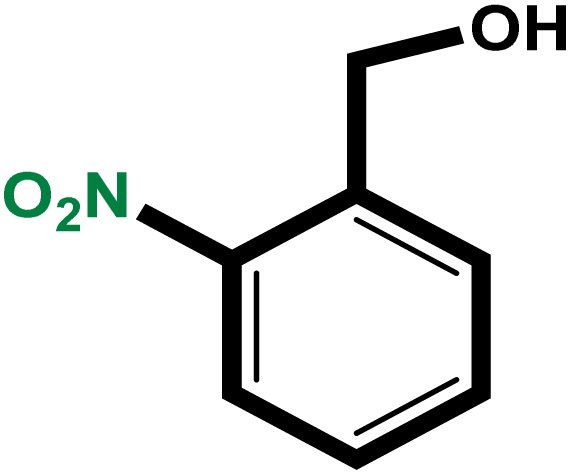	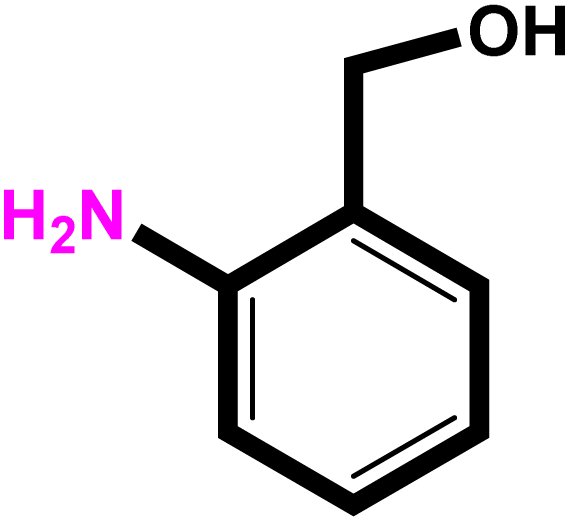	2j	1	95
9	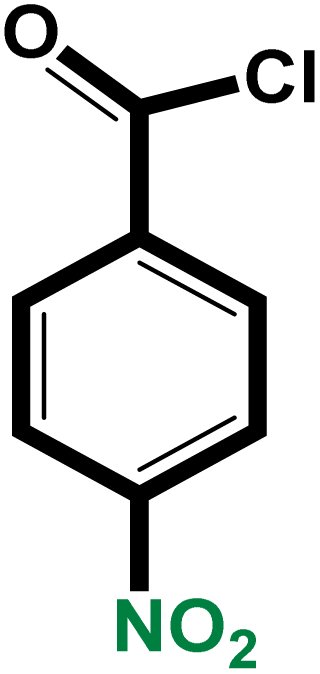	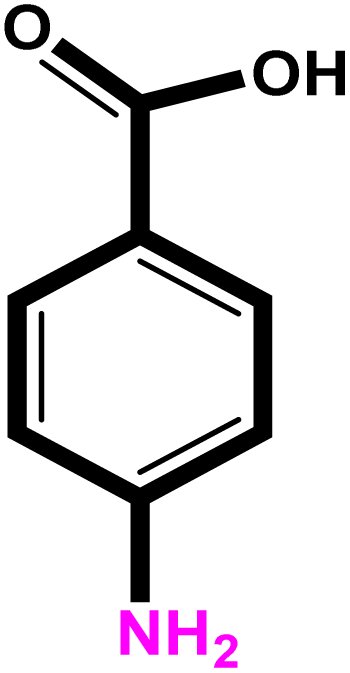	2k	1	70

a Reaction conditions: nitroarenes (0.5 mmol), NaBH_4_ (1.5 mmol), cat. (0.03 g), H_2_O (3 mL), 70 °C.

b Isolated yield.

#### One-pot reductive C–N cross-coupling reaction of various nitroarenes using NaBH_4_ catalyzed by Fe_3_O_4_@SiO_2_@OERT-Ni-Cu nanocatalyst

The C–N cross-coupling reaction of various nitroarenes is carried out using acrylonitrile (1.2 mmol) in H_2_O (3 mL) in the presence of NaBH_4_ (1.5 mmol) and a catalyst (30 mg; 0.3 mol% Ni, 0.33 mol% Cu) at 70 °C for a fixed duration, as shown in Scheme 5 and Table 3. The substrate scope was assessed by considering both the electronic and steric features of the substituents, as well as how they might interact with the catalytic system. Product 3a (70%), derived from nitrobenzene, was selected as the reference substrate because it does not contain additional substituents that could meaningfully alter the reaction. Under these conditions, substrates 3b, 3e, and 3i delivered comparatively high yields of 80%, 90%, and 83%, respectively. This high efficiency is likely due to the beneficial electronic effects imparted by their electron-withdrawing substituents, particularly when located at the *para* position, where nitro-group reduction can be promoted while steric crowding is kept to a minimum. The results obtained for products 3d, 3f, and 3h clearly reflect the influence of steric effects and substituent positioning. Although 3d (85%) was produced in good yield even in the presence of an *ortho* iodine substituent, indicating that the steric influence is reasonably tolerated, 3f (70%) showed only moderate performance when methyl and chloro substituents were introduced. In the case of 3h (40%), the lower yield may be attributed to the combined impact of *ortho* steric hindrance and the possible interaction of the hydroxyl group with the catalytic active sites. In contrast, products 3c and 3g afforded markedly lower yields of 30% and 25%, respectively. The poor yield of 3c can mainly be ascribed to the competing reduction of the aldehyde group under the reaction conditions, whereas the low performance of 3g may be related to coordination of the nitrile group to the Ni/Cu active sites, which could impede effective substrate activation. Overall, these observations show that reaction efficiency is governed not by one single structural parameter, but by the combined effects of electronic factors, steric accessibility, catalyst-substrate interactions, and competing side processes. This substrate-dependent behavior highlights the key role of both substituent identity and position in controlling the efficiency and selectivity of the catalytic C–N coupling reaction.

**Scheme 5:**
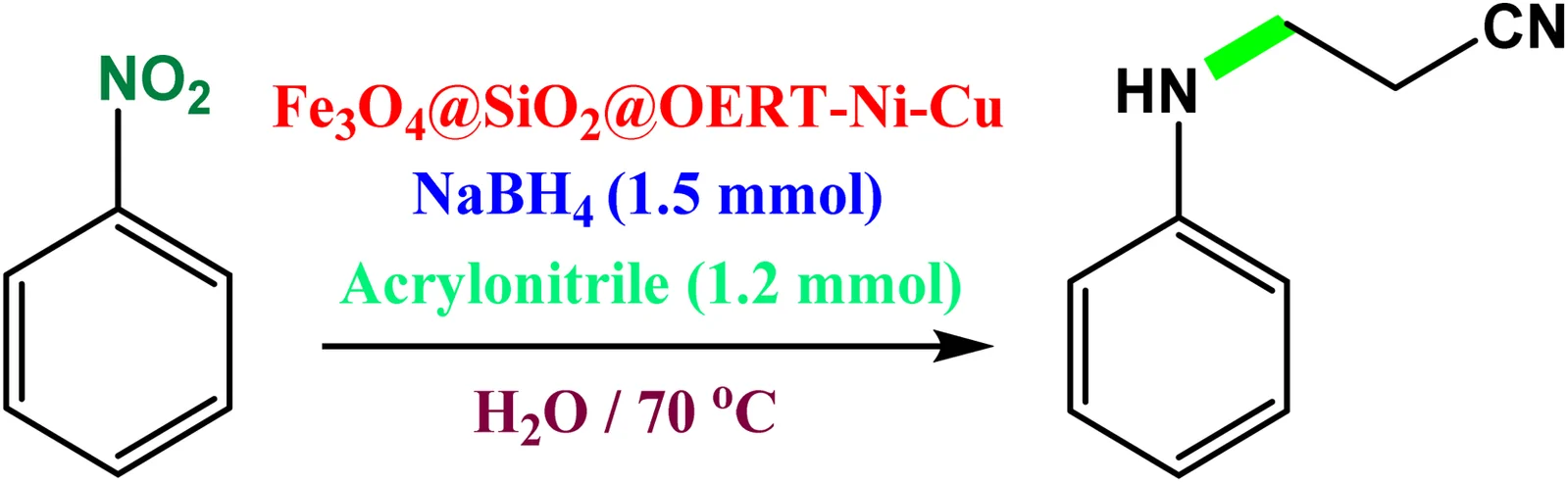
One-pot reductive C–N cross coupling of nitroarenes using NaBH_4_ catalyzed by Fe_3_O_4_@SiO_2_@OERT-Ni-Cu in water.

**Table 3:** One-pot reductive C–N cross-coupling of various nitroarenes using Fe_3_O_4_@SiO_2_@OERT-Ni-Cu^a^

Entry	Substrate	Product	Product no.	Yield^b^ (%)
1	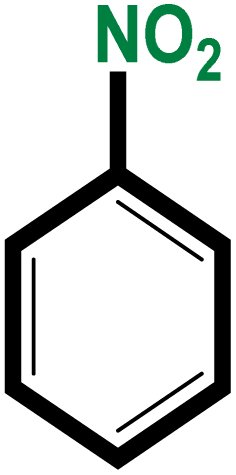	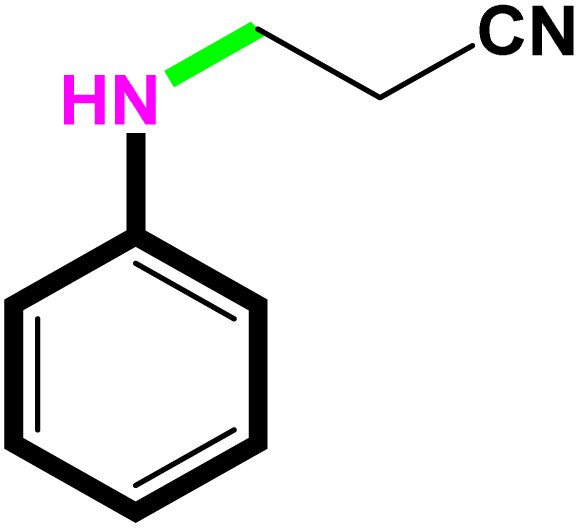	3a	70
2	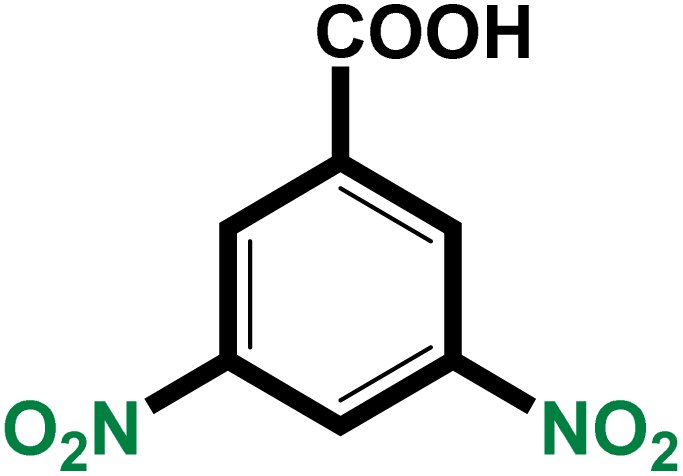	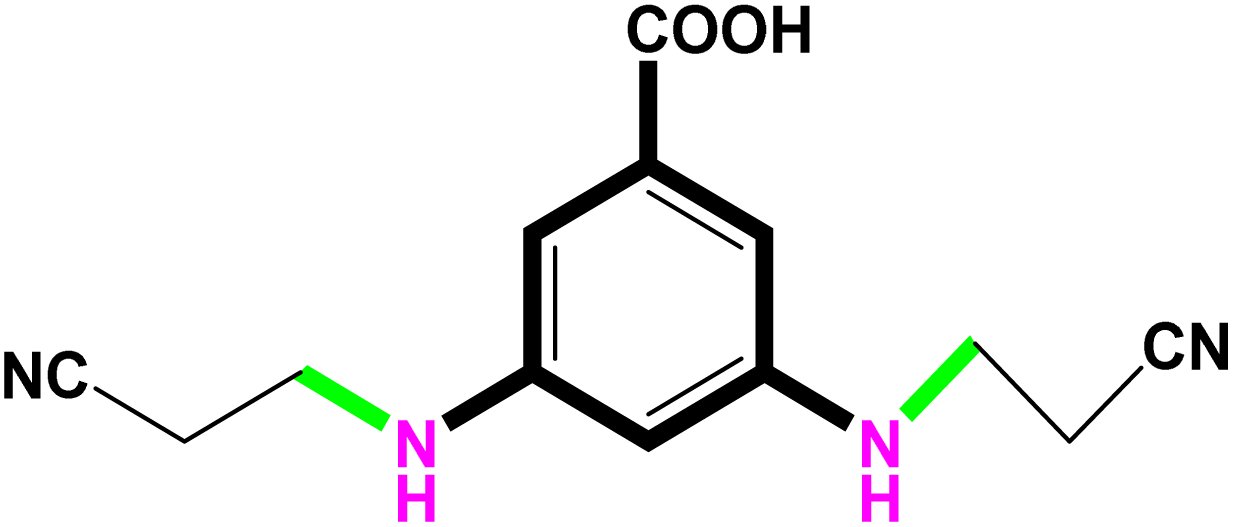	3b	80
3	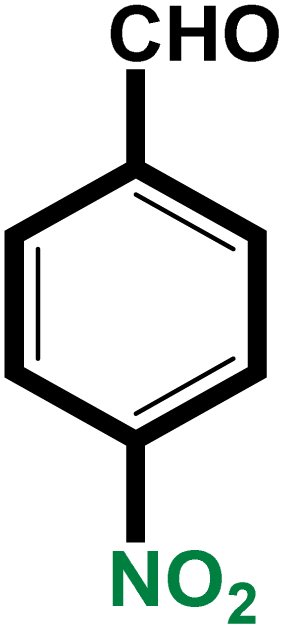	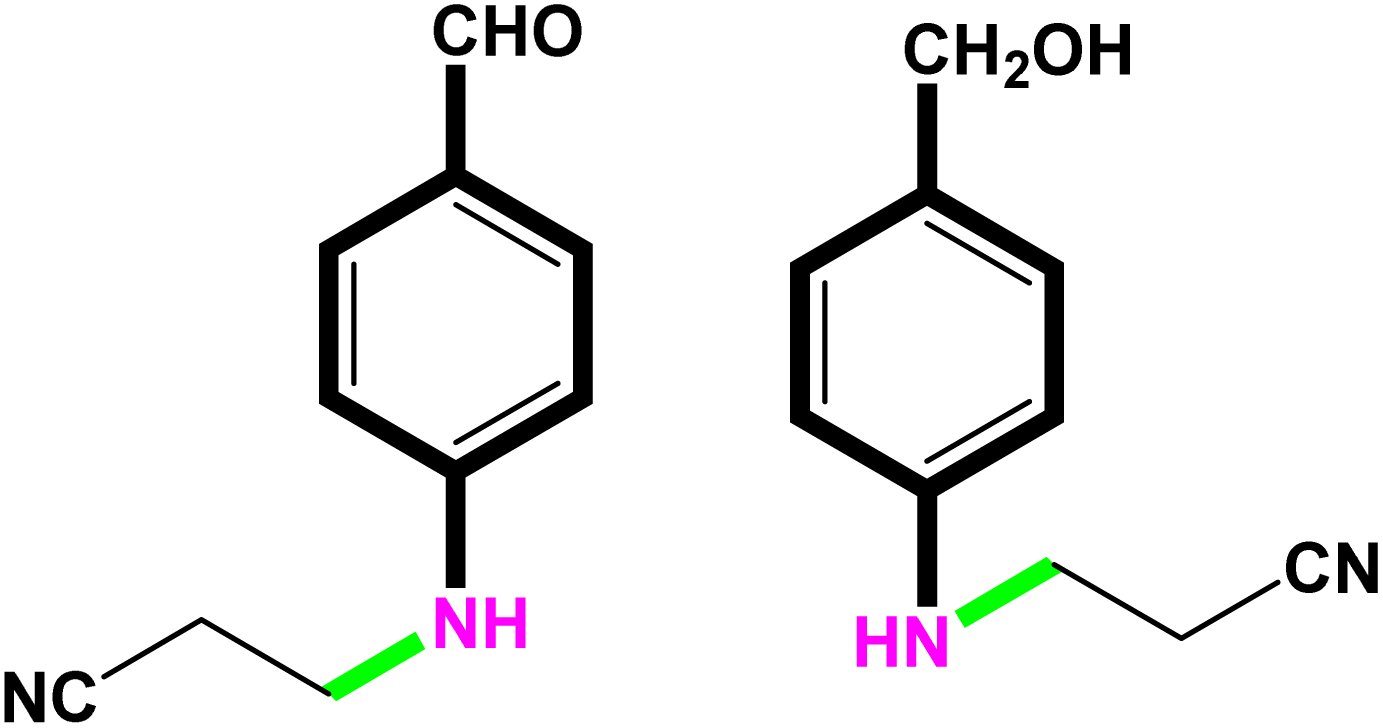	3c, 3d	30
4	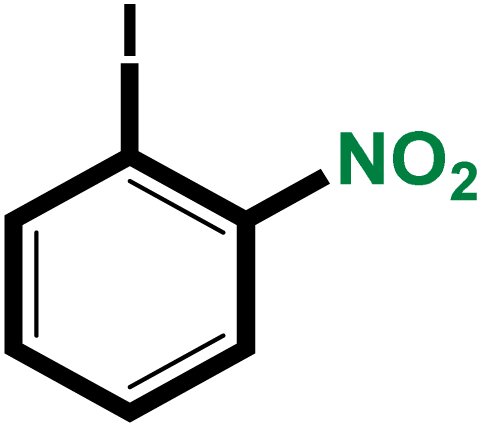	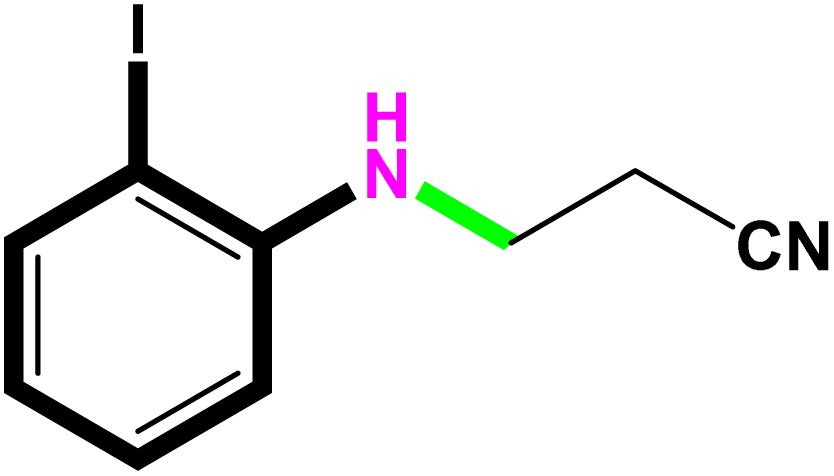	3e	85
5	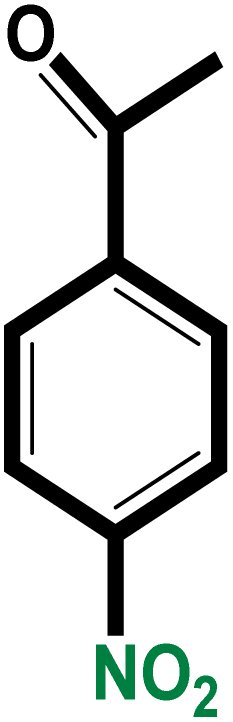	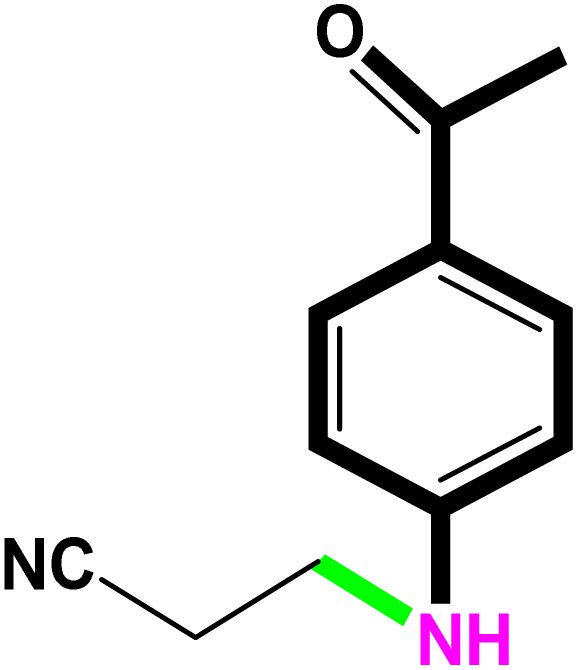	3f	90
6	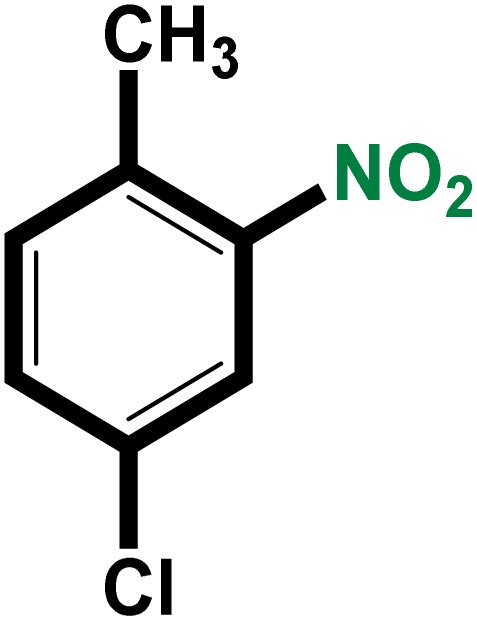	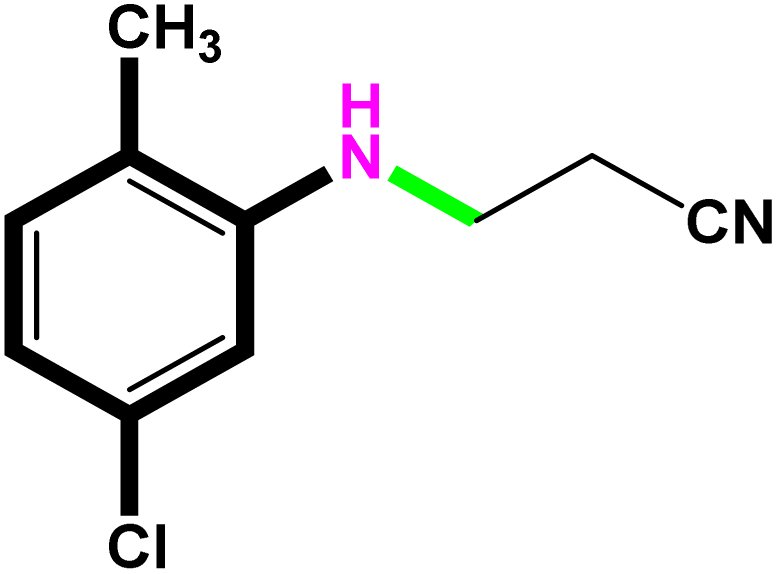	3g	70
7	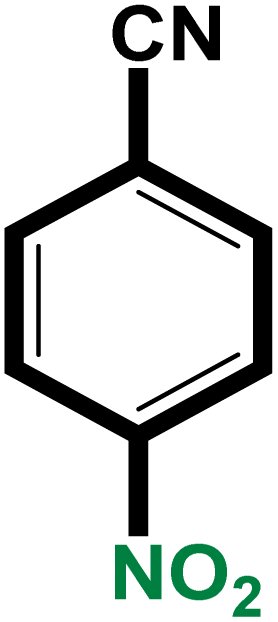	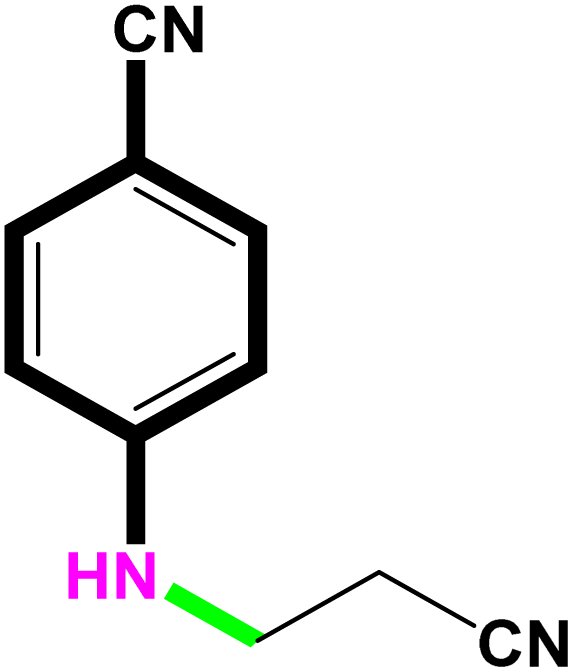	3h	25
8	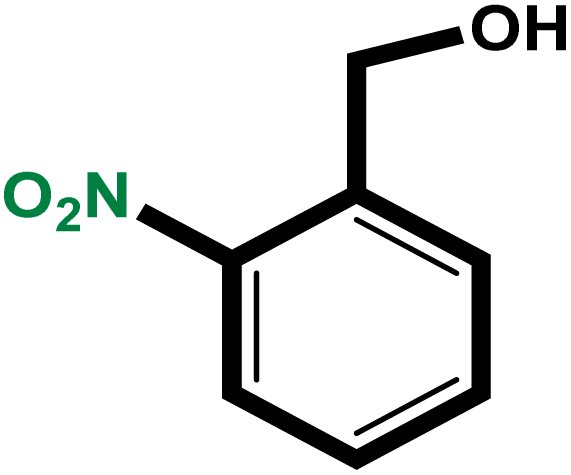	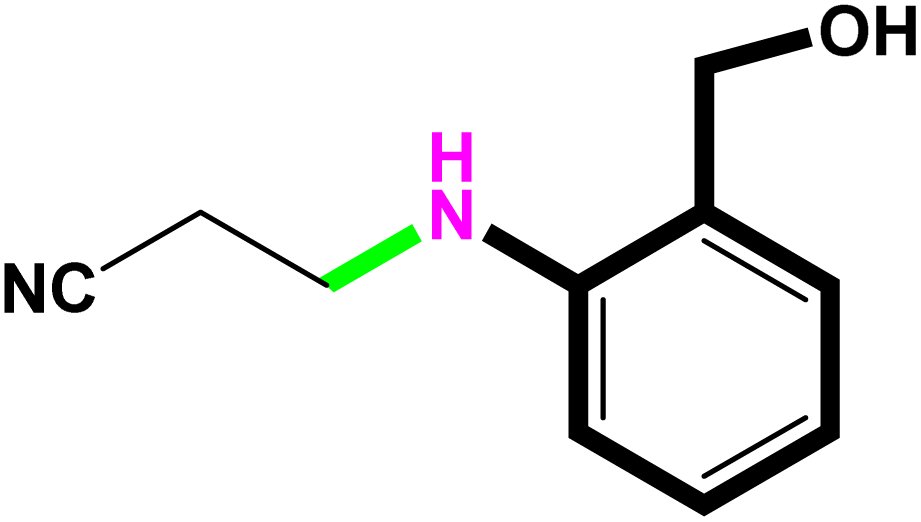	3i	40
9	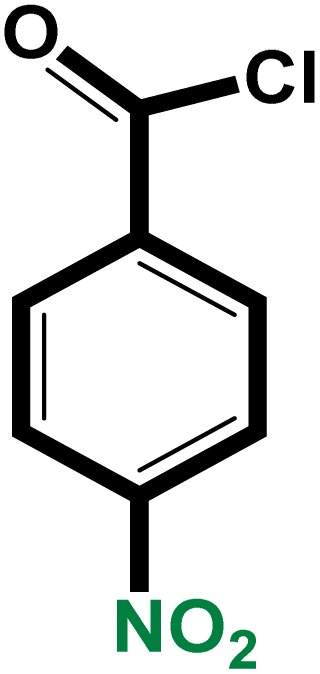	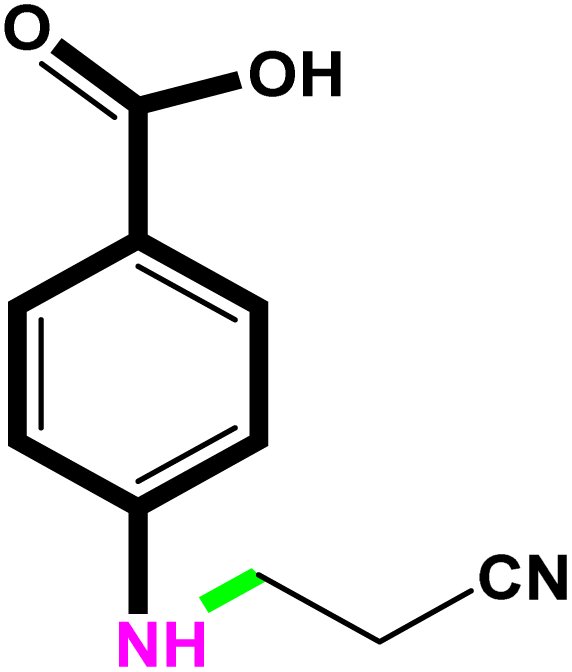	3j	83

a Reaction conditions: nitroarenes (0.5 mmol), NaBH_4_ (1.5 mmol), acrylonitrile (1.2 mmol), cat. (0.03 g), H_2_O (3 mL), 70 °C, 2 h.

b Isolated yield.

### Control experimental

The advantages and synergistic effects of the model reactions are investigated by introducing different catalyst components in control experiments. The results are presented in Table 4. From this table, it is evident that the reaction conditions do not produce any coupling product in the presence of Fe_3_O_4_ nanoparticles, Fe_3_O_4_@SiO_2_, Fe_3_O_4_@SiO_2_-NH_2,_ and OERT. Furthermore, the catalytic properties of nickel and copper salts are assessed, revealing no significant product formation in the reduction of nitrobenzene. In the next step, evaluation of the synergistic effect of the two metals shows that the catalytic activity is increased to 65% in the reduction reaction when only the nickel center is coordinated. By coordinating both copper and nickel metals to form a bimetallic catalytic system, the high catalytic efficiency is achieved in the reduction reaction. The reaction conducted in the absence of a catalyst does not yield any products, underscoring the essential role of the catalyst in the reduction of nitro compounds. The Fe_3_O_4_@SiO_2_@OERT-Ni-Cu nanocatalyst demonstrates superior performance compared to other catalysts due to its high efficiency, short reaction time, and ease of recycling.

**Table 4:** Designed control experiments for the reduction of nitroarenes

Entry	Catalyst^a^	Yield b(%)
1	Fe_3_O_4_ NPs	0
2	Fe_3_O_4_@SiO_2_	0
3	Fe_3_O_4_@SiO_2_-NH_2_	0
4	OERT	0
5	Fe_3_O_4_@SiO_2_-NH_2_-OERT	Trace
6	Fe_3_O_4_@SiO_2_@OERT-Ni	65
7	Fe_3_O_4_@SiO_2_@OERT-Ni-Cu	98
8	Ni(OAc)_2_·4H_2_O	35
9	Cu(OAc)_2_·4H_2_O	45
10	Without catalyst	0

a Reaction conditions: nitrobenzene (0.5 mmol), NaBH_4_ (1.5 mmol), cat. (0.03 g), H_2_O (3 mL), 70 °C, 1 h.

b Isolated yield.

Table 5 summarizes a comparison of various studies reported on the reduction of nitrobenzene. According to the results, the synthesized bimetallic nanocatalyst in the present study not only reduces the reaction time and requires a smaller amount of catalyst under favorable conditions but also demonstrates superior performance compared to many reported catalysts. This enhanced performance is attributed to its easy separation and recyclability using an external magnetic field.

**Table 5:** Summary of characteristics of the reviewed studies on the reported catalysts for the reduction of nitrobenzene

Entry	Catalyst (mg)	Reaction conditions	Time	Yield (%)	Ref.
1	Fe_3_O_4_@-SiO_2_@OERT-Ni/Cu (30)	H_2_O/NaBH_4_/70 °C	1 h	99	This work
2	Ni–Au/C (50)	EtOH, H_2_, 120 °C	3 h	100	29
3	Pd/IL (2.85)	Glycerol, H_2_, 80 °C	18 h	99	30
4	Ni–Zn/AC-350 (100)	DMF, H_2_, 80 °C	4 h	98.36	31
5	RHPrNH_2_@Ag (500)	H_2_O/NaBH_4_/reflux	55 min	99	32
6	Fe_3_O_4_@PAMAM/Ni(0)-*b*-PEG (4)	H_2_O/NaBH_4_/40 °C	2 h	91	33
7	Pd-DNA-Fe_3_O_4_ (18)	1,4-Dioxane, N_2_H_4_, 85 °C	6 h	90	34

### Mechanism study

The proposed mechanism for the reduction of nitroaromatic compounds to the corresponding amines is illustrated in Scheme 6. Sodium borohydride is adsorbed onto the surface of the nanocatalyst, where hydride molecules bind to the copper-nickel metals, resulting in the formation of NiCu–H species. In the next step, the hydrogens from the NiCu–H species are transferred to nitrobenzene, leading to the formation of nitroso intermediates. Subsequent hydride transfer to the nitroso intermediate produces hydroxylamine, and finally, the aniline product is obtained through the removal of water.

**Scheme 6:**
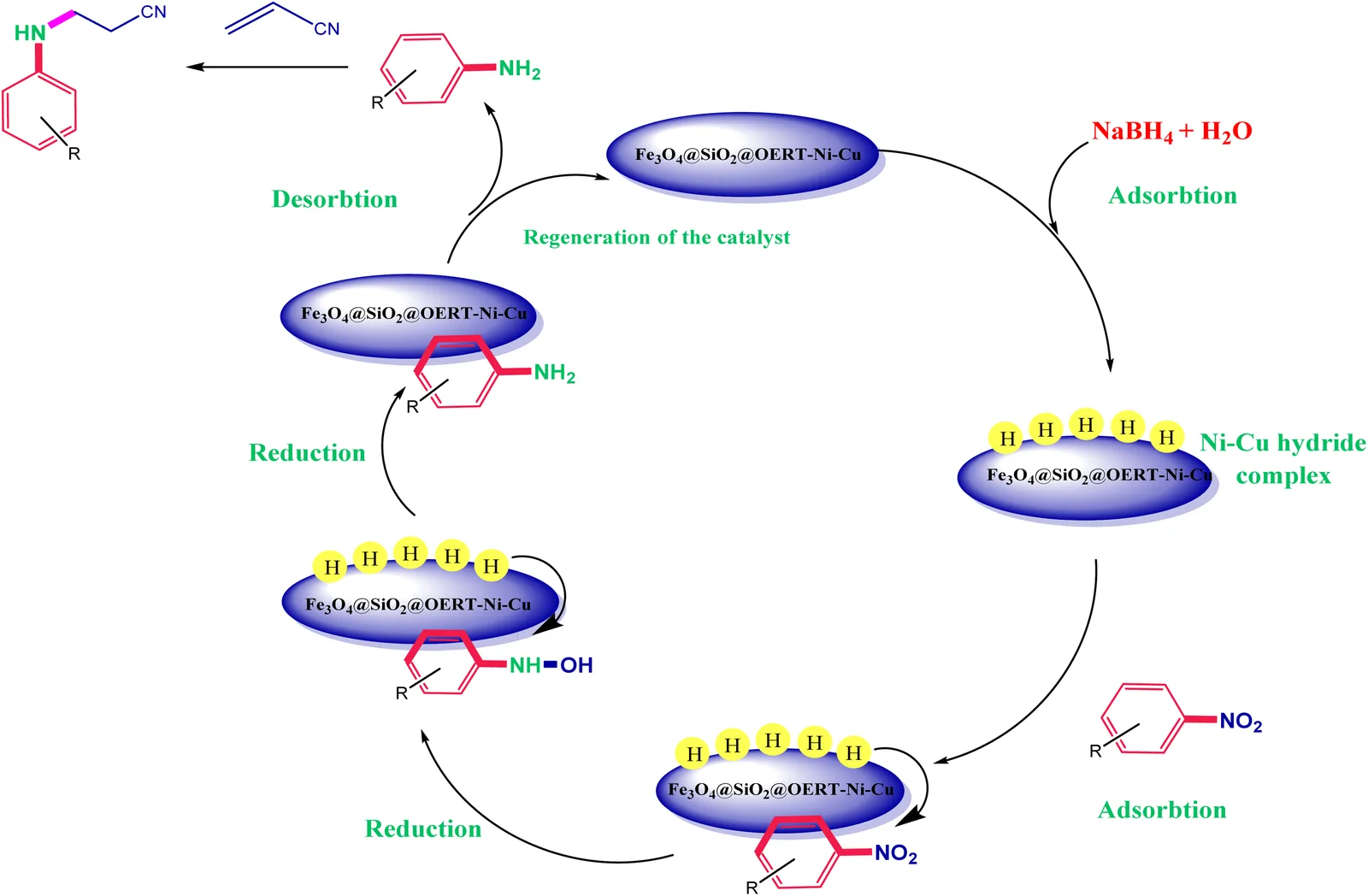
Proposed mechanism for the synthesis of amines using Fe_3_O_4_@SiO_2_@OERT-Ni-Cu as a nanocatalyst.

### Recyclability study

The most important feature of magnetic nanocatalysts is their ability to be recovered from the reaction medium and reused. Magnetic nanocatalysts can be easily collected using an external magnet for subsequent reactions. We have investigated the recovery of the synthesized Fe_3_O_4_@SiO_2_@OERT-Ni-Cu nanocatalyst in the reduction of nitrobenzene. The nanocatalyst is separated from the reaction medium with a magnet, washed with water and ethanol, and then dried in an oven. The results of FT-IR, TEM, and FE-SEM analyses confirm the stability and activity of the nanocatalyst after seven cycles (Fig. 7). The synthesized Fe_3_O_4_@SiO_2_@OERT-Ni-Cu nanocatalyst can be reused multiple times without significant loss of activity, making it both cost-effective and environmentally friendly.

**Fig. 7:**
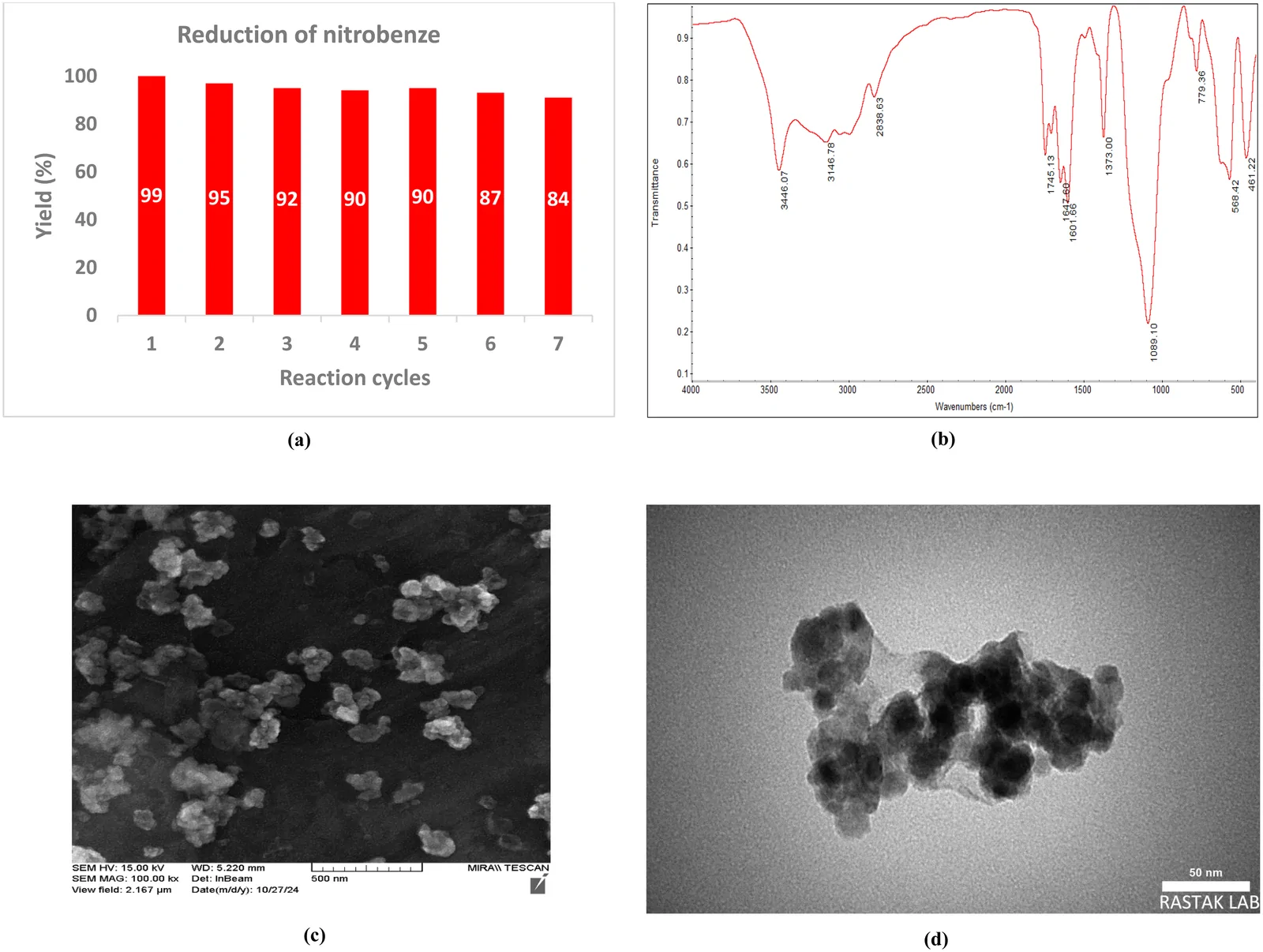
(a) Recovery and reusability of Fe_3_O_4_@SiO_2_@OERT-Ni-Cu nanocatalyst in the reduction of nitrobenzene reaction under optimized reaction conditions, (b) FT-IR spectrum, (c) FE-SEM, and (d) TEM images of the recovered catalyst after the 7th run, respectively.

## Conclusion

In this study, we designed a novel magnetic Fe_3_O_4_@SiO_2_@OERT-Ni-Cu bimetallic nanocatalyst, which is characterized using various techniques such as FT-IR, XRD, and VSM. The catalytic performance of the Fe_3_O_4_@SiO_2_@OERT-Ni-Cu nanocatalyst is evaluated in the reduction of nitroaromatic compounds and aromatic amines, as well as in the carbon-nitrogen coupling reactions derived from the reduction of nitroaromatic compounds. Under mild conditions, high product yields are obtained in the presence of the synthesized nanocatalyst. The nanocatalyst demonstrated excellent reusability and ease of separation from the reaction medium, indicating its high stability. Furthermore, the synergistic effect between the two metals results in superior catalytic activity of the bimetallic nanocatalyst compared to the monometallic nickel catalyst.

## Author contributions

Mansoore Shahabi: conceptualization, methodology, investigation, experimental work, data analysis, writing – review & editing, writing – original draft. Mohhammad Ali Naseri: supervision, project administration, validation investigation, writing – review & editing. Seyyedeh Ameneh Alavi G.: formal analysis, data curation, investigation, writing – review & editing. All authors have read and approved the final manuscript and agree with the order of authors listed in the manuscript. We also confirm that there are no disputes regarding authorship or the contributions of any of the authors.

## Conflicts of interest

The authors declare that they have no known competing financial interests or personal relationships that could have appeared to influence the work reported in this paper.

## Supplementary Material

RA-OLF-D5RA04730G-s001

## Data Availability

Data available on request from the authors. Supplementary information (SI) is available. See DOI: https://doi.org/10.1039/d5ra04730g.
