# Supplementary material for: Ni–Cu bimetallic nanocatalyst stabilized on magnetic nanoparticles for reduction of nitroarenes and one-pot C–N cross-coupling reactions
Source: RSC Adv. 2026 Sep 28;16(45):57890–902. doi: 10.1039/d5ra04730g (PMC13618067; doi:10.1039/d5ra04730g)

Supplementary Information (SI) for RSC Advances.
This journal is © The Royal Society of Chemistry 2026

Supplementary Materials

Ni-Cu Bimetallic Nanocatalyst Stabilized on Magnetic Nanoparticles for Reduction of Nitroarenes and One-Pot C-N Cross-Coupling Reactions

*Mansoore Shahabi, Mohammad Ali Nasseri, * Seyyedeh Ameneh Alavi G.*

Department of Chemistry, Faculty of Sciences, University of Birjand, P. O. Box 97175-615, Birjand, Iran.

**Corresponding author E-mail: manaseri@birjand.ac.ir*

^{1}H NMR characterization data of the Reduction of Nitroarenes products:

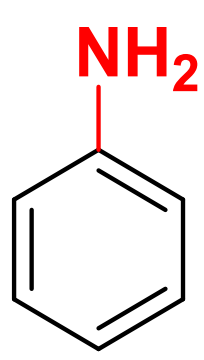

Aniline (Table 2, Entry 1). Colorless liquid. ^{1}H NMR (300 MHz, $CDCl_3$). δ (ppm): 7.32 (dd, *J* = 16.2, 8.6 Hz, 2H), 7.22 (d, *J* = 7.2 Hz, 1H), 7.11 (d, *J* = 7.6 Hz, 2H), 4.15 (s, 2H).

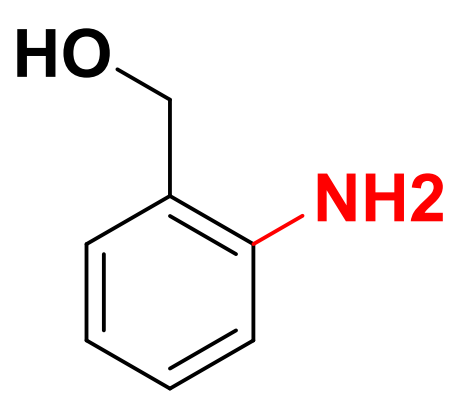

(2-aminophenyl)-methanol (Table 2, Entry 8). Off white solid. ^{1}H NMR (300 MHz, DMSO). δ (ppm): 7.04 (d, *J* = 7.4 Hz, 1H), 6.95 (t, *J* = 7.7 Hz, 1H), 6.60 (d, *J* = 8.0 Hz, 1H), 6.51 (t, *J* = 7.3 Hz, 1H), 5.06 – 4.98 (m, 1H), 4.91 (s, 2H), 4.37 (d, *J* = 4.5 Hz, 2H).

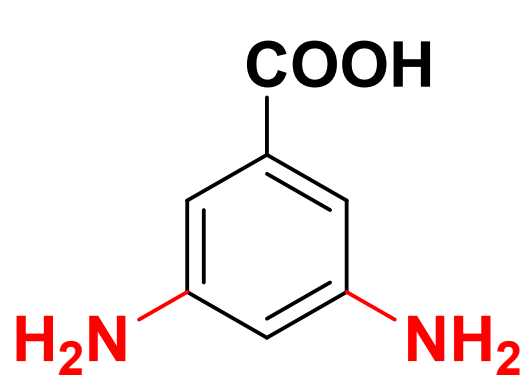

3,5-diaminobenzoic acid (Table 2, Entry 2). Brown solid. ^{1}H NMR (300 MHz, DMSO). δ (ppm): 6.44 – 6.37 (m, 2H), 5.99 (s, 1H), 5.02 (s, 4H).

^{1}H NMR spectra for the Reduction of Nitroarenes Compounds:

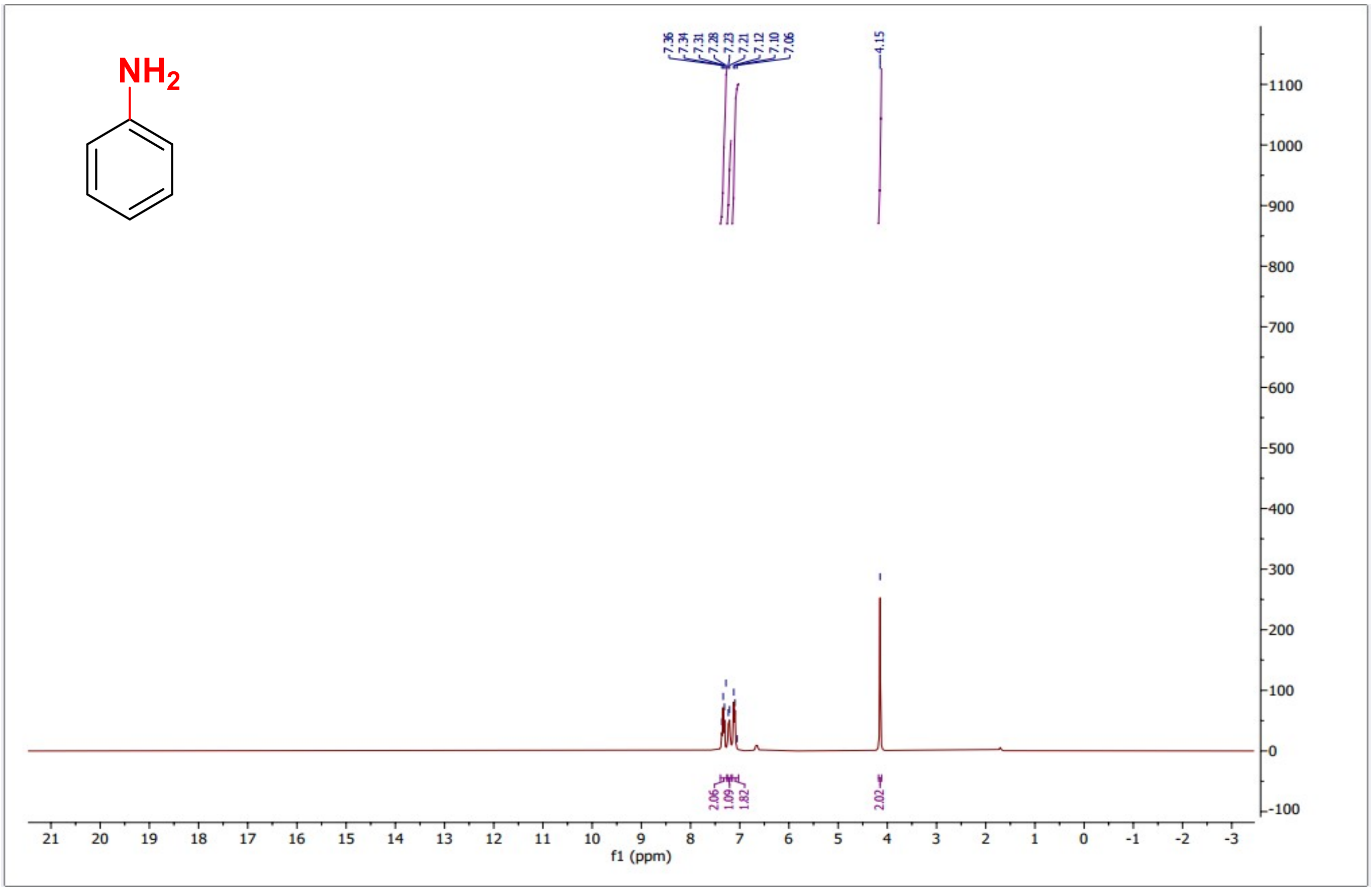

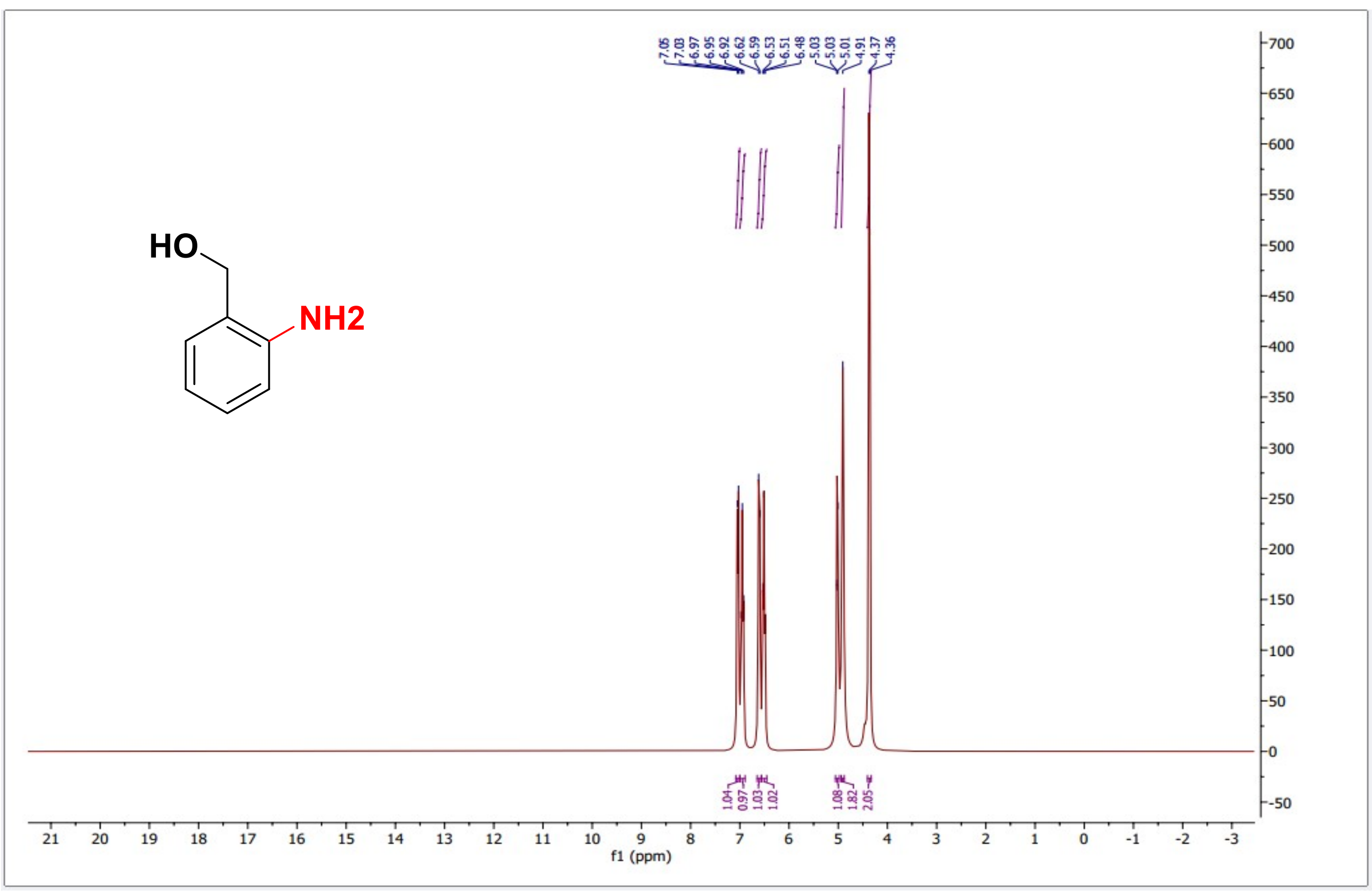

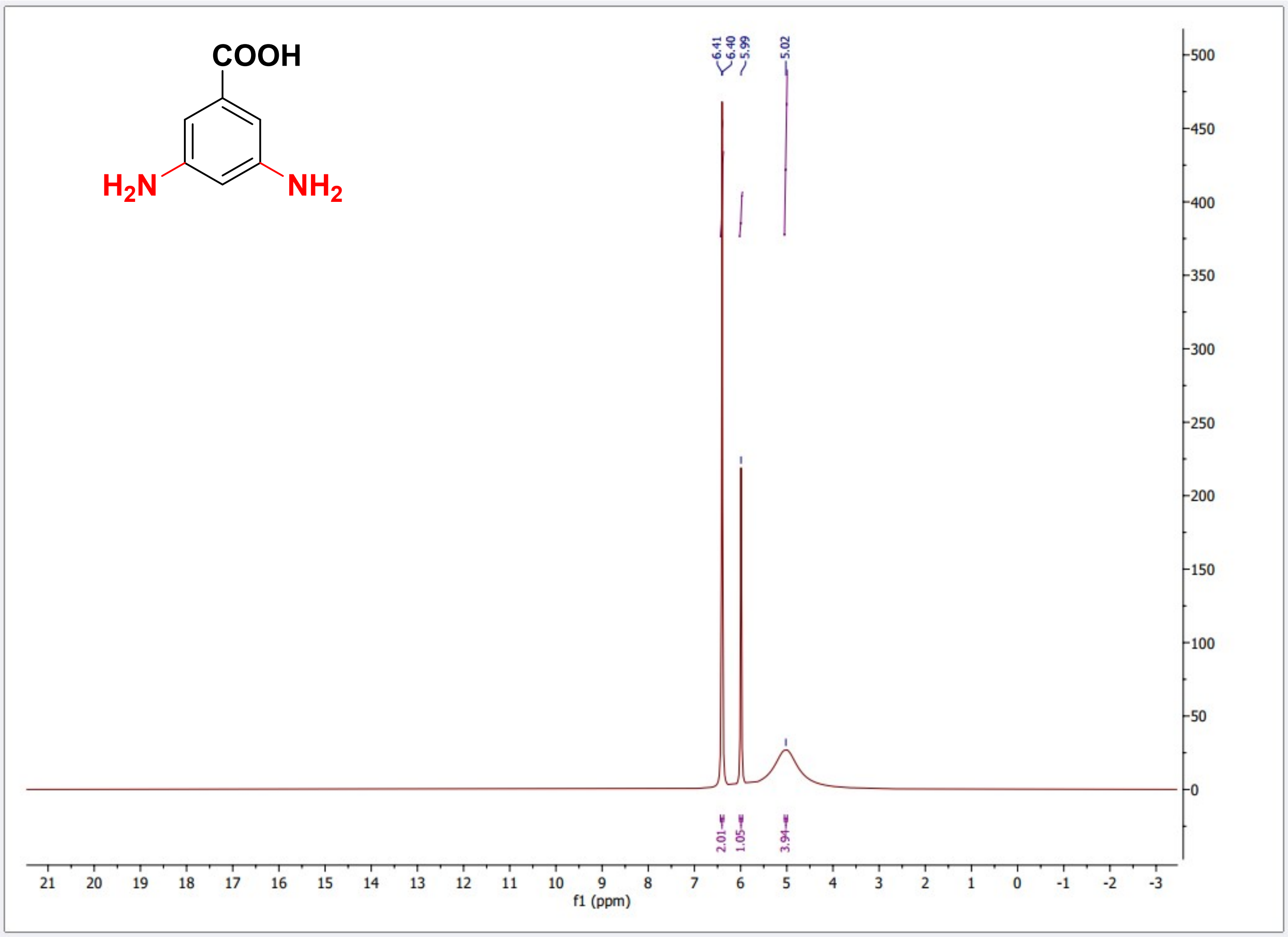

^{1}H NMR characterization data for One Pot C-N Cross Coupling Reactions product:

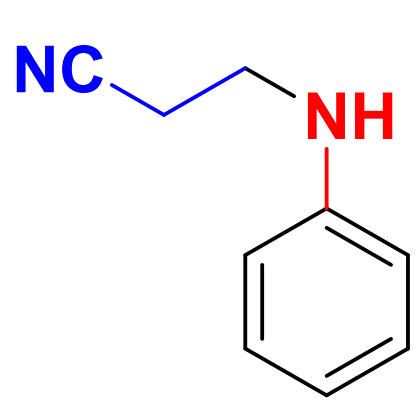

3-(Phenylamino)propanenitrile (Table 3, Entry 1): Brown colored solid. ^{1}H NMR (300 MHz, DMSO). δ (ppm): 7.09 (t, J = 7.7 Hz, 2H), 6.70 – 6.52 (m, 3H), 4.04 (s, 1H), 3.31 (t, J = 6.4 Hz, 2H), 2.71 (t, J = 6.5 Hz, 2H).

^{1}H NMR and FTIR spectra for One Pot C-N Cross Coupling Reactions product:

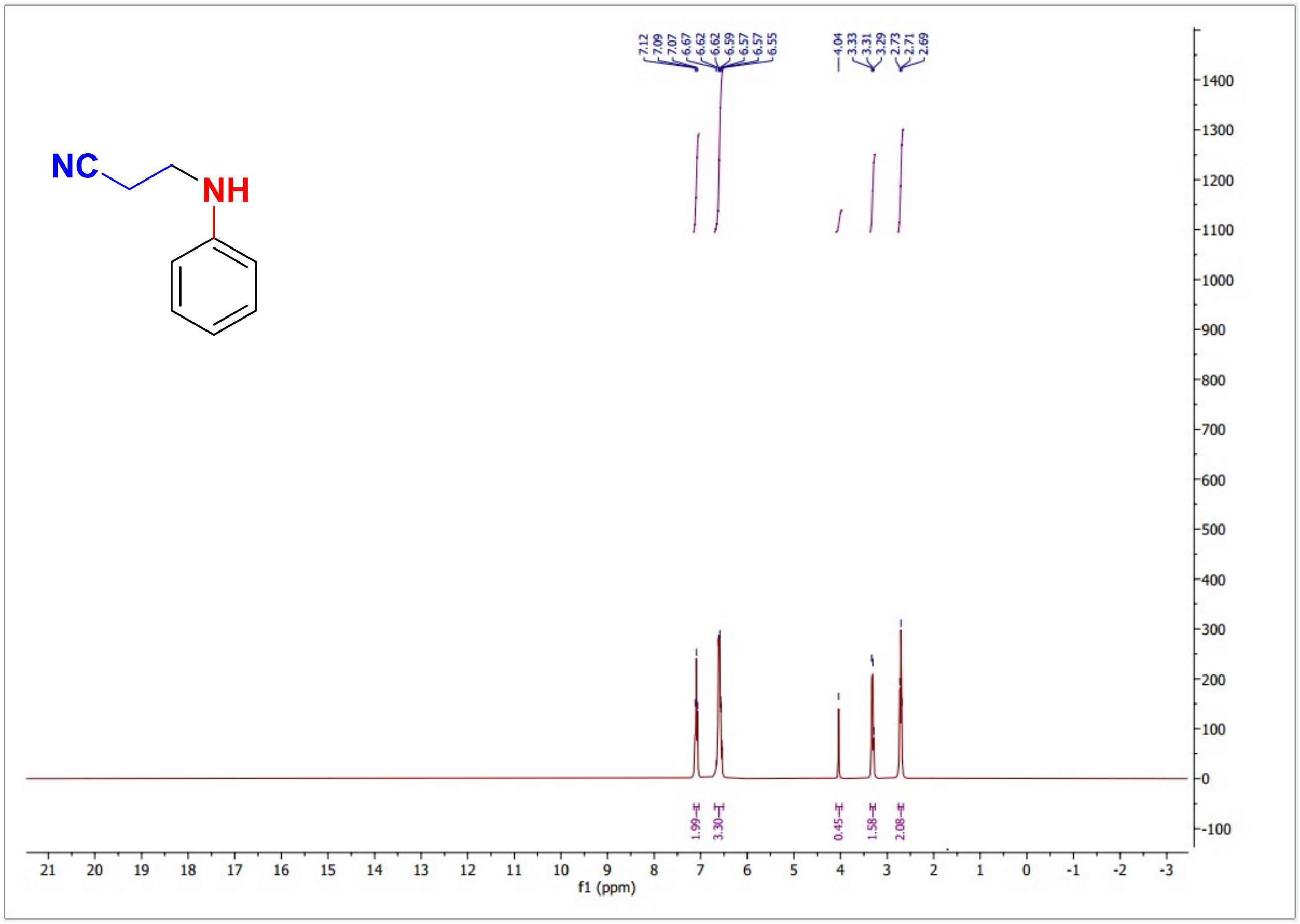

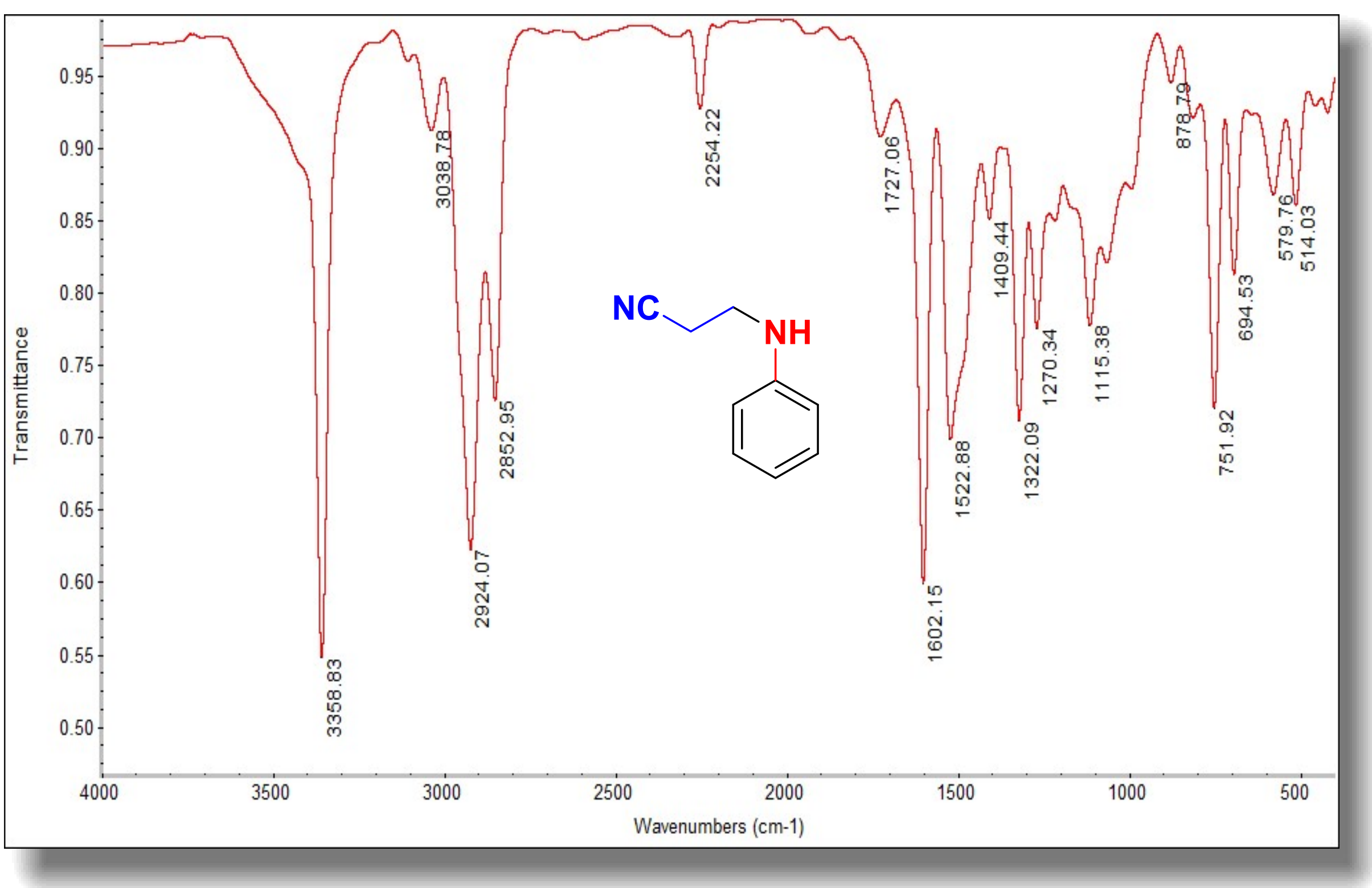

Supplement: Supplementary file 1 [file RA-016-D5RA04730G-s001.pdf]
